# Boron Cluster Anions Dissolve *En Masse* in Lipids Causing Membrane Expansion and Thinning

**DOI:** 10.1002/anie.202412834

**Published:** 2024-10-31

**Authors:** Andrea Barba‐Bon, Alae El Haitami, Coralie Pasquier, Ivana Nikšić‐Franjić, Olivier Diat, Pierre Bauduin, Sophie Cantin, Werner M. Nau

**Affiliations:** ^1^ School of Science Constructor University Campus Ring 1 28759 Bremen Germany; ^2^ Laboratoire de Physicochimie des Polymères et des Interfaces (LPPI) Université de Cergy-Pontoise 5 mail Gay Lussac F-95031 Cergy-Pontoise-Cedex France; ^3^ Institut de Chimie Séparative de Marcoule CNRS UMR 5257 CEA Université de Montpelier ENSCM F-30207 Bagnols sur Cèze Cedex France

**Keywords:** Cluster anions, Noncovalent interactions, Monolayers, Chaotropic effect, Superchaotropic ions

## Abstract

Boron clusters are applied in medicinal chemistry because of their high stability in biological environments and intrinsic ability to capture neutrons. However, their intermolecular interactions with lipid membranes, which are critical for their cellular delivery and biocompatibility, have not been comprehensively investigated. In this study, we combine different experimental methods – Langmuir monolayer isotherms at the air–water interface, calorimetry (DSC, ITC), and scattering techniques (DLS, SAXS) – with MD simulations to evaluate the impact of *closo*‐dodecaborate clusters on model membranes of different lipid composition. The cluster anions interact strongly with zwitterionic membranes (POPC and DPPC) via the chaotropic effect and cause pronounced expansions of lipid monolayers. The resulting lipid membranes contain up to 33 mol % and up to 52 weight % of boron cluster anions even at low aqueous cluster concentrations (1 mM). They show high (μM) affinity to the hydrophilic‐hydrophobic interface, affecting the structuring of the lipid chains, and therefore triggering a sequence of characteristic effects: (*i*) an expansion of the surface area per lipid, (*ii*) an increase in membrane fluidity, and (*iii*) a reduction of bilayer thickness. These results aid the design of boron cluster derivatives as auxiliaries in drug design as well as transmembrane carriers and help rationalize potential toxicity effects.

## Graphical abstract

Superchaotropic dodecaborate cluster anions adsorb efficiently on lipid mono‐ and bilayers, affecting the physical properties of the membrane more strongly than conventional chaotropic anions. Cluster immersion in the bilayer leads to a reorganization of the lipid chains, affording hybrid organic–inorganic membranes with expanded surface area per lipid as well as thinner membranes with increased fluidity.

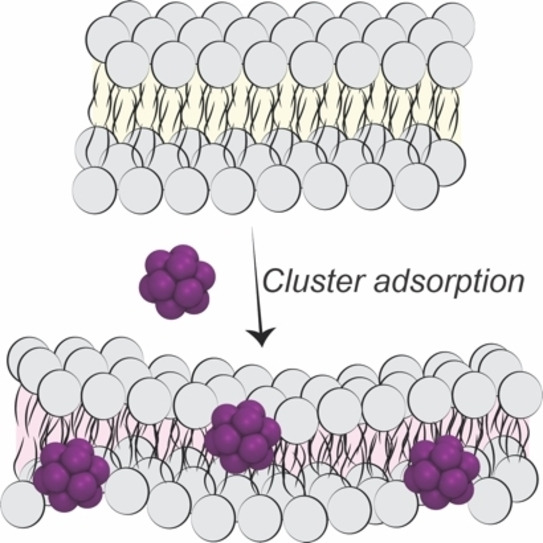

## Introduction

In contrast to organic molecules, which are generally limited by the tetravalency of carbon to aliphatic or planar aromatic frameworks, boron has an intrinsic propensity to undergo multivalent interactions and form three‐dimensional inorganic cage structures.[^1^, ^2^] An example is the icosahedral dodecaborate cluster di‐anion, B_12_H_12_^2−^, which displays 3D‐aromaticity^[3]^ with delocalized σ instead of π bonds. The resulting clusters are not only structurally appealing, but they show frequently an exceptional thermal and chemical stability^[4]^ that can exceed that of organic compounds by far.^[5]^ Because of this elevated stability, they have already found applications in diverse areas such as metal extraction,[^6^, ^7^] hydrogen storage,^[8]^ as well as catalysis,^[9]^ and they have been used for luminescent materials^[10]^ as well as ion‐selective electrodes.^[11]^

As man‐made compounds, boron clusters are not only abiotic in nature, but, in fact, resistant to bacterial and enzymatic degradation.^[15]^ This can result in long residence times in biological environments, which has attracted significant attention in medicinal chemistry.[^16^, ^17^, ^18^] Boron clusters have applications in boron neutron capture therapy,[^16^, ^19^, ^20^, ^21^, ^22^] antimicrobial[^23^, ^24^] as well as anticancer therapy,[^25^, ^26^] and cellular imaging.[^27^, ^28^] Recently, a new biological application of boron clusters has emerged, because the inorganic anions were shown to act as transmembrane carriers for a range of impermeable hydrophilic bioactive molecules (such as neutral or cationic peptides and pharmaceutical drugs), both in artificial membranes and living cells.[^29^, ^30^, ^31^, ^32^, ^33^] This new biological activity is associated with the superchaotropic nature of these very large charge‐delocalized cluster ions, which allows them to concomitantly interact with the cargo molecules as well as the lipid bilayer membranes.[^12^, ^13^, ^34^, ^35^]

**Figure 1:**
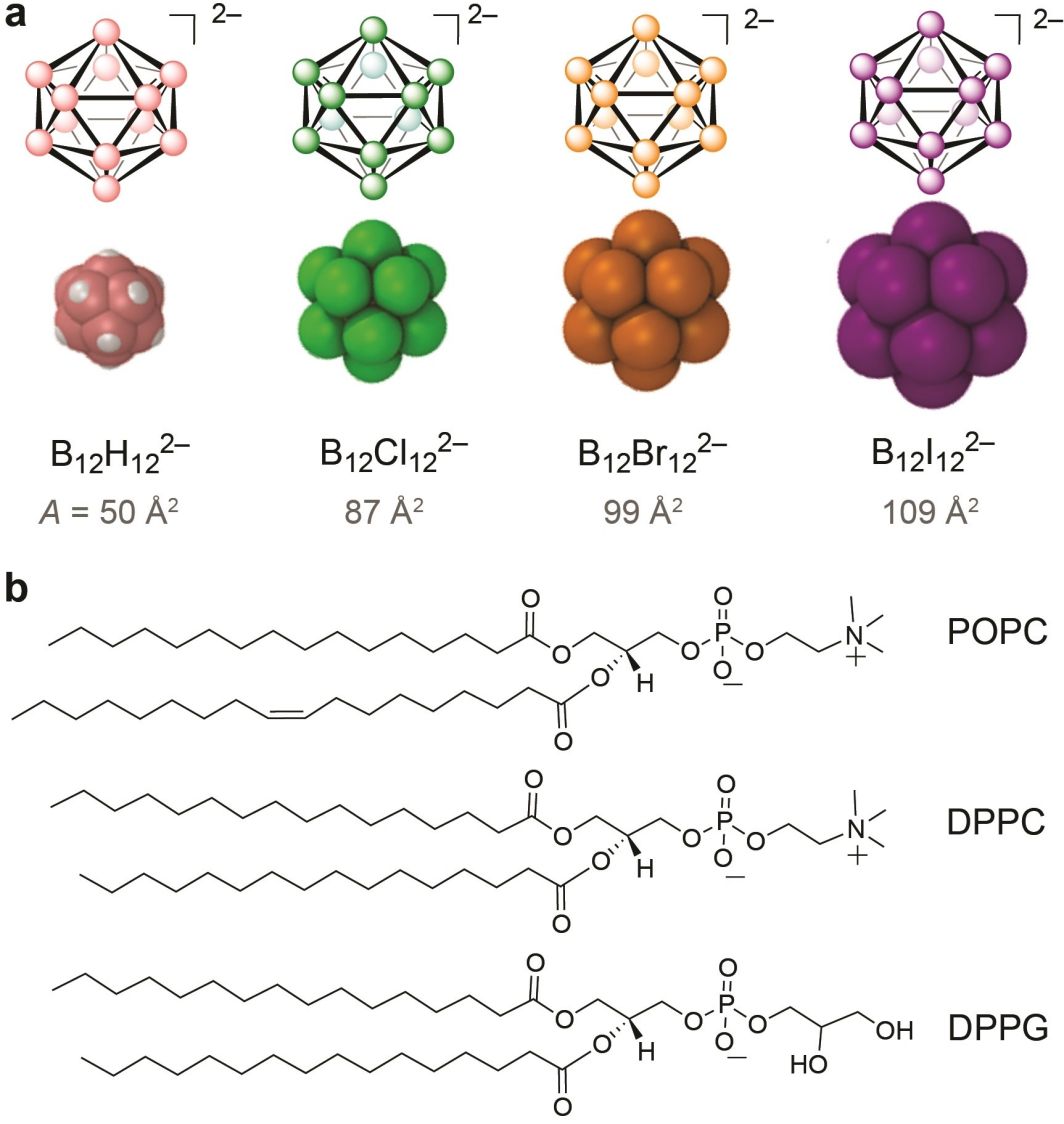
**a**) Chemical structures (top, 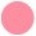
=BH, 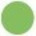
=BCl, 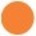
=BBr, and 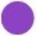
=BI) and space‐filling molecular models (bottom) of dodecaborate clusters with increasing cross‐sectional area (calculated from their maximal van‐der‐Waals radii).[^12^, ^13^, ^14^] **b**) Chemical structures of the phospholipids selected to evaluate cluster‐lipid interactions. POPC and DPPC both have the same zwitterionic head, but POPC possesses an unsaturated hydrocarbon chain. DPPG has the same saturated alkyl chains as DPPC but differs in its negatively charged head group.

The nature of the intermolecular interactions of boron clusters with membranes – which is likely key to the understanding of their permeability‐enhancing effects,[^15^, ^17^, ^22^, ^23^, ^24^, ^27^, ^28^, ^36^] their transmembrane carrier potential,[^29^, ^30^] as well as their membrane‐lytic activity[^29^, ^37^] – has not been studied in detail.[^26^, ^37^, ^38^, ^39^, ^40^, ^41^] To fill this gap, we investigate herein boron cluster‐membrane interactions by using the dodecaborate family (B_12_X_12_^2−^, X=H, Cl, Br, I) as prototype (Figure 1a). As membrane models we use Langmuir monolayers[^42^, ^43^, ^44^, ^45^] and large unilamellar vesicles (LUVs), as models of lipid bilayers.[^46^, ^47^, ^48^, ^49^]

We selected the following phospholipids to mimic different lipid types (Figure 1b): 1‐palmitoyl‐2‐oleoyl‐sn‐glycerol‐3‐phosphocholine (POPC), 1,2‐dipalmitoyl‐2‐sn‐3‐phosphocholine (DPPC), and 1,2‐dipalmitoyl‐sn‐glycero‐3‐phosphorylglycerol (DPPG). POPC and DPPC have been widely used as zwitterionic model membranes. They have very different phase transition temperatures and exist in different phases at 25 °C, which allows studies in dependence on membrane fluidity. The lipid with anionic charge (DPPG) was included to allow us to identify the role of electrostatic interactions. To understand the cluster‐lipid interactions, we measured Langmuir monolayer isotherms at the air–water interface and performed differential scanning calorimetry (DSC), isothermal titration calorimetry (ITC), dynamic light scattering (DLS), and small‐angle X‐ray scattering (SAXS) experiments, supported by MD simulations.

## Results and Discussion

### Effect on Langmuir Monolayers

**Figure 2:**
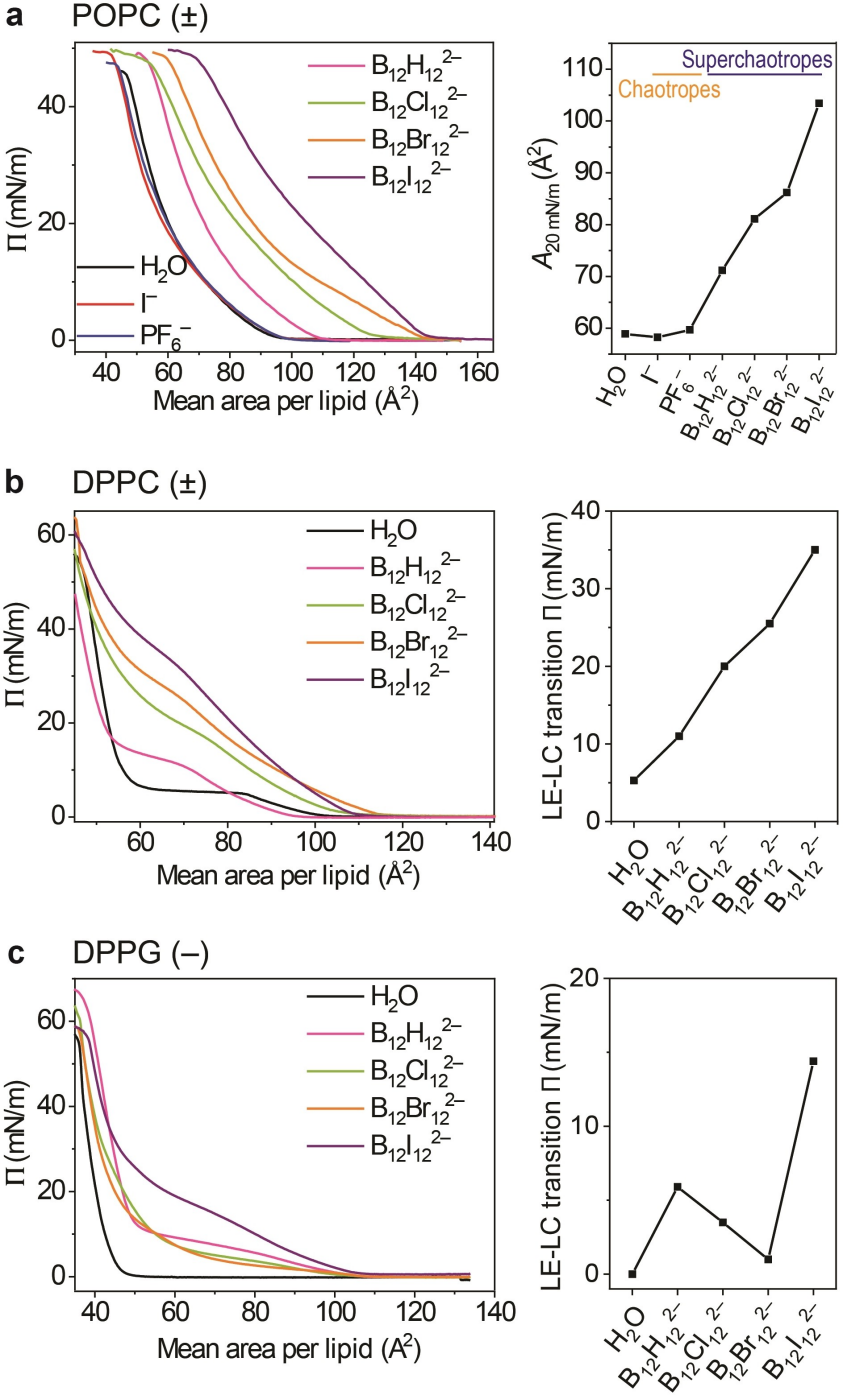
Cluster‐membrane interactions of different phospholipid monolayers on subphases containing chaotropic anions (1.0 mM). **a**) Left: π‐A isotherms for POPC monolayers on a subphase with different anions. Right: Mean area per molecule at a constant surface pressure of 20 mN/m in dependence on increasing chaotropicity (from left to right). **b**) Left: π‐A isotherms for DPPC monolayers on a subphase with different boron clusters. Right: LE‐LC transition surface pressure in dependence on increasing chaotropicity (from left to right). **c**) Left: π‐A isotherms for DPPG monolayers on a subphase with different boron cluster solutions. Right: LE‐LC transition surface pressure as a function of increasing chaotropicity (from left to right).

The cluster effect on air–water monolayers was evaluated first, at 20 °C. Figure 2a shows the pressure‐area (π‐A) isotherms for POPC monolayers formed on different subphases. POPC monolayers over pure water are known to remain in the liquid‐expanded (LE) phase (for schematic illustrations of the different phases see Scheme S1), regardless of surface pressure, until collapse occurs at ∼43 mN/m; the extrapolated area per lipid molecule is 67 Å^2^ (Figure S1), as reported.[^50^, ^51^] When boron clusters were present in the subphase below the POPC monolayer (1.0 mM), the monolayer was strongly expanded, with the apparent surface area per lipid increasing roughly with the chaotropicity of the cluster (Figure 2a) and, expectedly, concentration (Figure S2 and S3). The observed expansion effects for POPC are exceptionally large (almost doubling of the area per lipid) for the most chaotropic clusters (B_12_Br_12_^2−^ and B_12_I_12_^2−^) and were detectable already at micromolar concentrations (e.g., at 50 μM for B_12_Br_12_^2−^, Figure S4). In contrast, conventional chaotropes (I^−^ and PF_6_^−^) showed no significant expansion of the monolayer at 1.0 mM concentrations (Figure 2a). It transpires that Langmuir monolayer experiments can be used as a sensitive tool to quantify the chaotropicity of cluster ions on account of their strong adsorption, an observation that is supported by previous sets of experiments for polyoxometalates,^[52]^ another class of superchaotropic anions.[^13^, ^53^, ^54^, ^55^, ^56^]

As can be seen from Figure 2a, the areas of the POPC monolayer increase by ca. 15 % for B_12_H_12_^2−^ to 80 % for B_12_I_12_^2−^ upon addition of 1.0 mM cluster anion. Although a precise analysis of the excess free energies and areas of mixing cannot be performed – because the clusters are insufficiently surface‐active to form monolayers on their own – the observed expansions are exceptionally large. By using the cross‐sectional areas of the quasi‐globular clusters (Figure 1a) along with the extrapolated area per POPC lipid, and by assuming no excess areas of mixing, one can estimate a cluster content between 17–33 mol % or a lipid:cluster ratio of ca. 5 : 1 (B_12_H_12_^2−^) down to 2 : 1 (for B_12_I_12_^2−^). This signifies that the superchaotropic anions act as true components in binary monolayers rather than as additives. In fact, when molecular weights are considered for the heavy clusters, mass compositions of 32 % and 52 % result for B_12_Br_12_^2−^ and B_12_I_12_^2−^, respectively, allowing the designation of the resulting assemblies as a new type of hybrid organic–inorganic POPC‐cluster monolayers.

Figure 2b shows the π‐A isotherms for DPPC monolayers formed on different subphases. On a water surface, DPPC monolayers show the well‐known surface‐pressure plateau at around 6 mN/m, characteristic of the first‐order transition from the liquid expanded (LE) to the liquid condensed (LC) phase[^57^, ^58^] (Scheme S1), in which the lipid molecules are closely packed and lose mobility. Addition of boron clusters of varying chaotropicity to the subphase (1.0 mM) yields a systematic shift of the LE‐LC transition to higher surface pressure; a similar behavior is observed for a temperature increase of a DPPC monolayer over pure water.^[59]^ The surface pressure at which the LE‐LC phase transition occurs increases significantly with the chaotropicity of the cluster (Figure 2b, right panel).

The observed effects for boron clusters exceed the previously observed salt effects on DPPC monolayers by far. For example, to reach a similar effect (phase transition starting at 11 mN/m) as for B_12_H_12_^2−^, the least chaotropic ion in the cluster series, a much higher concentration of conventional chaotropic anions is required (∼0.1 M of I^−^).[^60^, ^61^, ^62^] Noteworthy, for the most chaotropic halogenated boron clusters, the LE‐LC phase transition reaches 35 mN/m, which would be similar to a temperature increase from 20 °C to around 38 °C over pure water, a temperature close to the critical point (44 °C).^[59]^ Such pronounced effects cannot be achieved with classical chaotropes. The experiments for DPPC reveal that the boron clusters are strongly adsorbed by the monolayers and stabilize the LE phase at higher surface pressures, which in turn can be interpreted as an increase in monolayer fluidity or “melting away” of the LC phase.

Langmuir isotherms for DPPG monolayers on different subphases (pure water and 1.0 mM dodecaborate clusters) are shown in Figure 2c, recorded at 20 °C. Expectedly, due to electrostatic repulsion between the negatively charged lipids and the cluster dianions, the effects were smaller than for the zwitterionic lipids. Note that the triple point temperature of anionic DPPG over water lies at 23 °C^[63]^ and that the gas phase transforms directly into the LC phase upon compression below this temperature. Above 23 °C, DPPG monolayers show an LE phase between the gaseous and LC phases.^[42]^ The phase behavior over pure water at 20 °C shows the known phase transition from the gas to the LC phase at 43 Å^2^.^[64]^ In the presence of boron clusters, a surface pressure plateau appears, characteristic of the LE‐LC phase transition occurrence (see Figure 2c, left panel). As shown in Figure 2c, right, the surface pressure at which the phase transition is detected is low for the less chaotropic ions in the series (only 1 mN/m for B_12_H_12_^2−^) while a significant effect is observed for the most chaotropic B_12_I_12_^2−^ cluster (LE‐LC phase transition at around 14 mN/m). Similar to DPPC, the effect of the dodecaborates on the DPPG monolayers was comparable to the effect of increasing temperature.^[42]^ For B_12_I_12_^2−^, the LE‐LC transition pressure would correspond to an increase in temperature by more than 15 °C.^[42]^ As was the case for DPPC monolayers, the adsorption of the clusters onto DPPG tends to stabilize the more fluid LE phase.

### Effect on Lipid Bilayer Surface Charge

**Figure 3:**
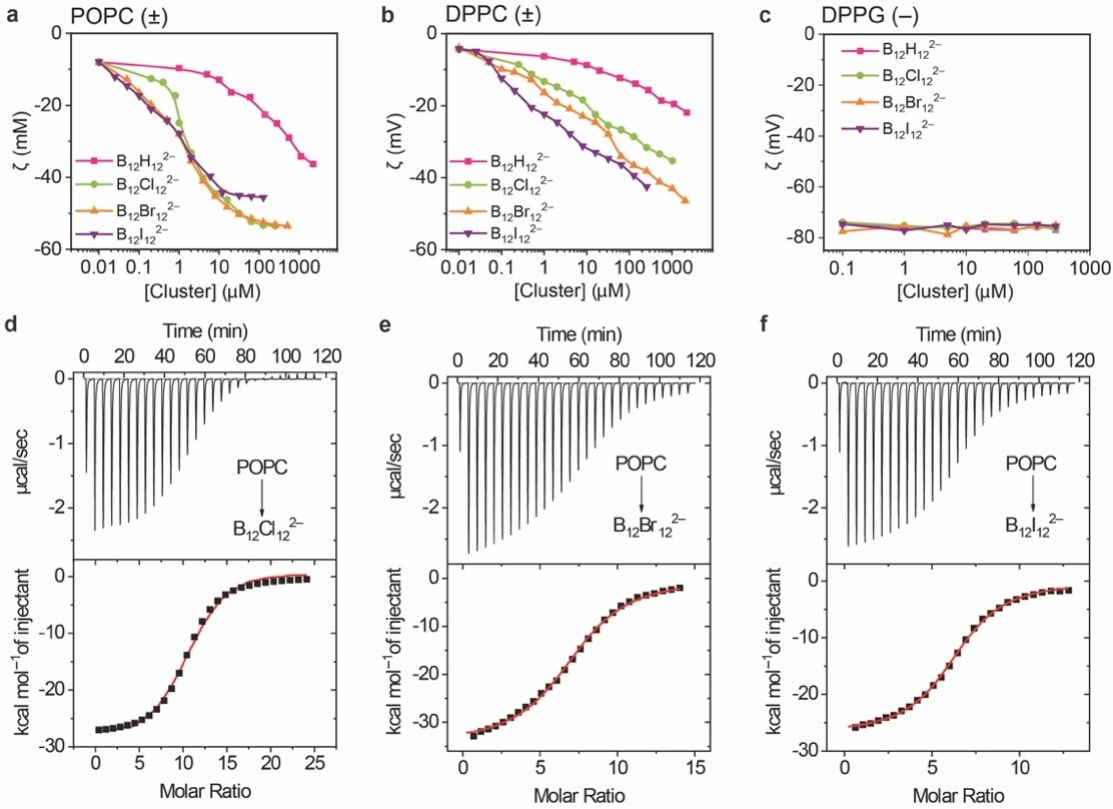
Top: *ζ*‐Potential measurements of **a**) POPC, **b**) DPPC, and **c**) DPPG liposomes (250 μM phospholipids in 10 mM Tris buffer, pH 7.4) as a function of boron cluster concentration after incubation with the clusters for 2 hours at 25 °C. Bottom: Microcalorimetric titrations of POPC liposomes with **d**) B_12_Cl_12_^2−^, **e**) B_12_Br_12_^2−^, and **f**) B_12_I_12_^2−^. Raw ITC data (upper panels, “direct” titrations) for sequential injections of the liposomal suspensions to the boron cluster solutions, and apparent reaction heats obtained from the integration of the microcalorimetric traces (lower panels). All experiments were conducted in 10 mM Tris buffer, pH 7,4. Lipid/cluster concentrations in mM: **d**) 2.6/0.023; **e**) 2.5/0.038; **f**) 3/0.05.

Liposomes – large unilamellar vesicles – were used next as a more advanced lipid bilayer model of the biological membrane, and changes on their *ζ*‐potential were followed in the presence of the clusters. Both zwitterionic liposome types (POPC and DPPC) show a *ζ*‐potential close to 0 in the absence of the clusters due to charge neutralization. The incubation with clusters led to negative *ζ*‐potentials for both POPC and DPPC (Figure 3a,b), revealing an interaction between the clusters and the lipid bilayer membrane. However, no significant changes in the liposomal size (measured by DLS, Figures S6–S8) were noticeable, except for B_12_I_12_^2−^ at high concentration (>300 μM), where membrane lysis was observed.[^29^, ^37^] In all experiments, the employed concentrations were chosen sufficiently low to bypass this side reaction.

It is known that conventional “small” chaotropic anions such as ClO_4_^−^ induce changes in the *ζ*‐potential of zwitterionic membranes, but very high concentrations (∼0.25 M) are required to achieve moderate shifts by 10 mV.^[65]^ We found that these effects were much exaggerated when we used the superchaotropic anions (Figure 3a). Even for the least chaotropic dodecaborate cluster, B_12_H_12_^2−^, only 20 μM are required to decrease the *ζ*‐potential by the same amount for POPC liposomes (Figure 3a); for the larger clusters (B_12_Br_12_^2−^ and B_12_I_12_^2−^) even 100 nM were sufficient to produce a decrease by ~20 mV for POPC, suggesting a more than 6 orders of magnitude higher affinity of the cluster anions than that of conventional chaotropes, as assessed by the concentration differences (nM versus mM).

Regarding the lipid type, the absolute changes in *ζ*‐potential were largest for POPC. For DPPC, ca. 10 times higher concentrations (1 μM) were required to afford the same effect as for POPC at 25 °C (Figure 3b). This result pointed to stronger interactions of the clusters with POPC membranes, presumably due to the different phase of DPPC liposomes at ambient temperature. Note that DPPC shows a gel‐to‐liquid phase transition around 41 °C.^[66]^ Indeed, when the experiments were performed at 50 °C (Figure S9, for B_12_Br_12_^2−^), where both POPC and DPPC are in the same (liquid) phase, both zwitterionic lipids showed a similar decrease in *ζ*‐potential. Regardless of the lipid type, the changes in *ζ*‐potential in dependence on the type of boron cluster correlated with the trends in chaotropicity, except that B_12_I_12_^2−^ showed a slightly smaller effect than B_12_Br_12_^2−^ at high concentrations (>10 μM). For DPPG, the ζ‐potential remained constant in the presence of all boron clusters (∼75 mV, Figure 3c), suggesting the absence of a sizable change in surface charge.

The absolute decrease in surface charge at ca. 100 μM cluster concentrations (Figure 3) amounts to ca. −25 mV for B_12_H_12_^2−^ and −50 mV for B_12_Br_12_^2−^. When comparing these values with the surface charge of the DPPG liposomes of similar hydrodynamic size (−75 mV) and neglecting differential counterion, double layer, as well as inner‐leaflet effects, but correcting for the di‐anionic character of the clusters, one can estimate between 15–33 mol % cluster adsorption, in a similar range as the estimate from the monolayer experiments. Evidently, liposomes of a new chemical composition and with distinct structural and electrical properties are being formed, formulated from lipid:cluster anion mixtures in molar ratios of 6 : 1 to 2 : 1. This compatibility is surprising from a structural point of view, because the mixing defies shape matching between the elongated lipids and the globular ions.

### Cluster Affinity to Bilayers

ITC experiments were performed to quantify the thermochemistry of boron cluster association to liposomes. The calorimetric profiles (Figure 3d,e,f and Figure S10–S14) reveal strong reversible interactions (>10^5^ M^−1^) between the halogenated clusters and the zwitterionic lipids in their fluid phase (POPC at 25 °C and DPPC at 50 °C). These values are 3 orders of magnitude larger than the affinities previously estimated for conventional chaotropic anions with POPC lipids (I^−^: 32 M^−1^, ClO_4_^−^: 115 M^−1^).^[67]^ The μM affinities rationalize the strong adsorption into the lipid monolayers and bilayers at mM and lower aqueous‐phase cluster concentrations (see above). The binding was found to be invariably an exothermic, enthalpically driven process (Table 1, S1, and S2). This is in line with the chaotropic effect as driving force for association of the anionic clusters to hydrophobic interfaces,^[13]^ which extends from dodecaborates to polyoxometalates.[^52^, ^53^, ^54^, ^55^, ^56^, ^68^, ^69^]. This thermochemical fingerprint has been originally observed for the binding of boron clusters to the hydrophobic cavities of cyclodextrins.[^12^, ^70^, ^71^, ^72^] Important to note, the “complexation” enthalpies of the clusters with the lipids (Δ*H*=−32±5 kcal mol^−1^) exceed those observed for cyclodextrins[^12^, ^70^, ^71^, ^72^] by far, suggesting a deep immersion to achieve effective desolvation.

**Table 1:** Binding constants (*K*_a_) and thermochemical data for the interaction of dodecaborate clusters with POPC liposomes at 25 °C by ITC.^[a]^

boron cluster	*n*	*K*_a_ (10^5^ M^−1^)^[b]^	Δ*H* (kcal/mol)^[b]^	*T*Δ*S* (kcal/mol)^[b]^
B_12_H_12_^2−^	n.h.^[b]^	n.h.	n.h.	n.h.
B_12_Cl_12_^2−^	10.4	4.1	−27.8	−20.2
B_12_Br_12_^2−^	7.3	5.3	−34.5	−26.6
B_12_I_12_^2−^	6.5	6.2	−27.1	−19.1

[a] Measured by “direct” titrations (POPC liposomes added to cluster) in 10 mM Tris buffer, pH 7.4, see Table S1 for additional data from “reverse” titrations. [b] n.h.: No reaction heat was obtained. Error in data is 10 % for the *n* values and *K*_a_, and ±0.5 kcal mol^−1^ for Δ*H* and *T*Δ*S* (SD, duplicates).

The trend in affinities and the complexation stoichiometries from the ITC data is more difficult to interpret. On one hand, the hydrogenated cluster showed no enthalpically detectable interaction at the employed 500 μM cluster concentrations, while the affinities of the three halogenated clusters were all high but showed only a small increase with chaotropicity (from 4.1×10^5^ M^−1^ for B_12_Cl_12_^2−^ to 6.2×10^5^ M^−1^ for B_12_I_12_^2−^), see Table 1 and S1. On the other hand, the stoichiometry of binding does not systematically increase with the size of the clusters (as intuitively expected if one assumes that larger clusters could be surrounded by more lipids), but rather with the inverse of the cluster size. Accordingly, the average number of lipids that a cluster is interacting with decreases from 10.4 (for B_12_Cl_12_^2−^) to 7.3 (B_12_Br_12_^2−^) to 6.5 (B_12_I_12_^2−^). These values fall nicely into the stoichiometric range independently estimated from the monolayer area expansions and the surface charges (see above). Conversely, assuming that a POPC liposome has a limited capacity to incorporate boron clusters, it would be able to adsorb 50 % more of the larger B_12_I_12_^2−^ than of the smaller B_12_Cl_12_^2−^, which in turn could be a reason for the higher membrane‐lytic effect of the former (see above).

Lastly, boron clusters showed a stronger affinity to the fluid‐phase zwitterionic membranes than to the same lipid in the gel (ordered) phase (DPPC liposomes, Figure S11 and S12), presumably because the fluid liquid phase can adapt better geometrically to the immersion of the clusters than the more rigid gel phase. No reaction heat was detected by ITC upon addition of the boron clusters to the anionic DPPG lipid bilayer either (Figure S13 and S14). This is in line with the *ζ*‐potential measurements (see above), which jointly suggest the absence of significant interactions for DPPG bilayers – even if the monolayer experiments pointed to an incipient interaction (see above).

### Effect on Membrane Fluidity

The preferential formation of the “more fluid” liquid expanded phase in the presence of boron clusters that we observed for the monolayer experiments manifested itself also in the phase transitions for lipid bilayer membranes as measured by DSC (Figure S15, Table 2). In the absence of clusters, DPPC liposomes show a pre‐transition peak at 34.9 °C and a main transition at 41.7 °C.[^66^, ^73^, ^74^] In this main transition (gel‐fluid phase transition) the lipid “melts” from the ordered gel phase to the disordered fluid phase (Scheme S2). The pre‐transition peak of DPPC was lowered when the liposomes were incubated with B_12_H_12_^2−^ (equimolar lipid:cluster concentrations), and completely disappeared in the presence of the halogenated derivatives. The main transition temperature decreased strongly in the presence of clusters, revealing an increase in membrane fluidity, in line with the general trend that the main phase transition of lipids is affected by chaotropic ions.[^75^, ^76^] The “melting effect” was most pronounced for B_12_Br_12_^2−^ and B_12_I_12_^2−^, which reduced the phase transition temperature by almost 5 °C (Table 2). It is noteworthy that much lower anion concentrations of the boron clusters (<500 μM) than for conventional chaotropic ions (0.1–3 M) are required to cause stronger shifts of the transition temperature.^[76]^ In addition, an increase in cooperativity (lower *σ* values, Table 2) was found for the two largest boron clusters, which can be due to a better lateral coupling of the lipids when they are “complexed” by the clusters; note that ITC revealed that one cluster can bind between 6–11 lipids (see above).

**Table 2:** Transition temperatures (*T*_m_), calorimetric (Δ*H*_t_) and van′t Hoff (Δ*H*_vH_) enthalpies,^[a]^ cooperativity parameter (*σ*), and the size of cooperative unit (C.U.) of phase transition of DPPC liposomes (500 μM) in the presence of dodecaborate anions (500 μM) obtained by DSC.

boron cluster	*T*_m_ (°C)	Δ*H*_t_ (kcal/mol)^[b]^	Δ*H*_vH_ (kcal/mol)^[b]^	*σ* (10^−4^)^[c]^	C.U.^[c]^
none	41.7	6.21	331	3.52	53.3
B_12_H_12_^2−^	41.5	6.32	315	4.03	49.8
B_12_Cl_12_^2−^	39.1	6.49	327	3.94	50.4
B_12_Br_12_^2−^	37.1	6.95	394	3.11	56.7
B_12_I_12_^2−^	36.7	5.39	417	1.67	77.4

[a] Data fitted with the Levenberg‐Marquardt non‐linear least‐square method, model 2 (non‐two‐state, see Methods). [b] 5 % error (SD, duplicates). [c] 5 % error (SD, calculated by considering error propagation with respect to both, Δ*H*_vH_ and Δ*H*_t_).

The effect of cluster concentration on the DSC thermograms was evaluated for B_12_Br_12_^2−^; the pre‐transition disappeared completely even at the lowest cluster concentration (100 μM), the main transition phase shifted to lower temperatures and the cooperativity increased proportionally with cluster concentration (Figure S15c and Table S3). In contrast, when DPPG liposomes were incubated with equimolar concentrations of the boron clusters, no significant changes of the native phase transition temperature (41 °C)[^77^, ^78^, ^79^] were observed (Figure S15).

### Effect on Membrane Structure

**Figure 4:**
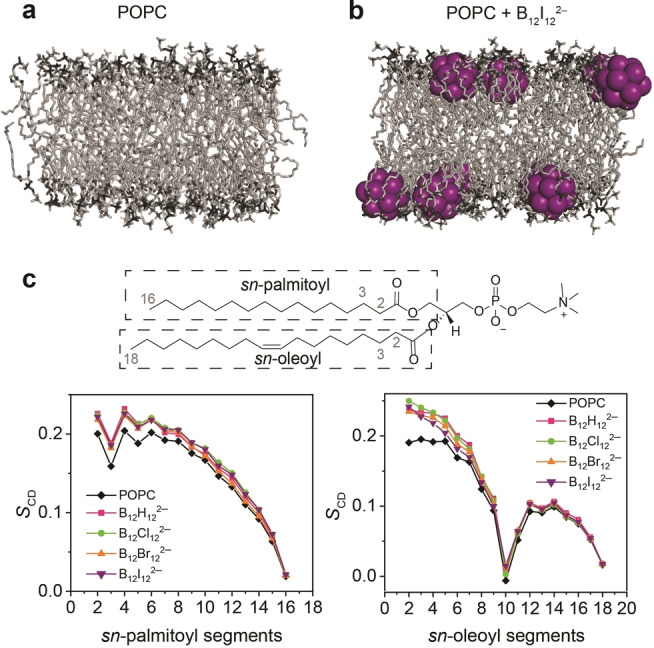
MD simulation snapshots (at 500 ns, 30 °C) of a neat POPC lipid bilayer and **b**) in the presence of B_12_I_12_^2−^; polar heads, dark grey; hydrophobic tails, light grey; B_12_I_12_^2−^, purple; water and sodium counter‐cations are not shown for clarity. **c**) POPC chemical structure (top) and simulated *S*_CD_ values (bottom) *vs* carbon segment number for the acyl chains of POPC alone (black line) and in the presence of clusters. The left panel represents the *sn*‐palmitoyl chain and the right panel the *sn*‐oleoyl chain.

We also carried out MD simulations, after first‐time parameterization of the halogenated dodecaborate clusters,[^80^, ^81^, ^82^] see Methods, and Table S4. We chose a lipid:cluster ratio of 128 : 10. This is slightly below the saturation points afforded by ITC measurements (*n* values, Table 1), comparable to the concentration regime of most experiments, but sufficiently large to produce sizable effects in the simulations. The MD simulations (Figure 4b and Figure S16) revealed an adsorption of the boron clusters onto the POPC membrane, with the tendency and depth of immersion increasing in the order B_12_H_12_^2−^<B_12_Cl_12_^2−^<B_12_Br_12_^2−^<B_12_I_12_^2−^. This confirmed the experimentally observed high affinity of the boron clusters with the lipid bilayer as well as the trend with cluster size. In fact, the clusters immersed fully into the headgroup region of the lipids and became localized below the zwitterionic groups but above the hydrophobic aliphatic region (Figure 4b and Figure S16–S17).[^68^, ^83^]

To obtain insight into the atomistic restructuring of the POPC lipid bilayer upon boron cluster adsorption, we extracted the lipid‐order parameter, *S*_CD_, see Figure 4c. This parameter reports on the global structure of the membrane and the variability of the atomic positions of the lipid tails in the membrane,[^84^, ^85^, ^86^] more positive *S*_CD_ values reflecting a more rigid orientation of the methylene C−H bonds orthogonal to the bilayer normal. As can be seen from Figure 4c, the *S*_CD_ values increased upon cluster addition for both, the palmitoyl and the oleoyl chain in the lipid bilayer, reflecting an increased structuring of the lipid chains. Although the variations between the different cluster types were not pronounced, the effect of boron cluster inclusion was largest for the methylene groups closer to the headgroups (C2‐C7), where the clusters are localized (Figure 4b). This is in contrast to the immersion of hydrophobic additives^[87]^ or the hydrophobic residues of amphiphilic additives,^[88]^ which affect mainly or equally the lipid order in the center of the membrane (C11‐C17).

### Effect on Membrane Thickness

**Figure 5:**
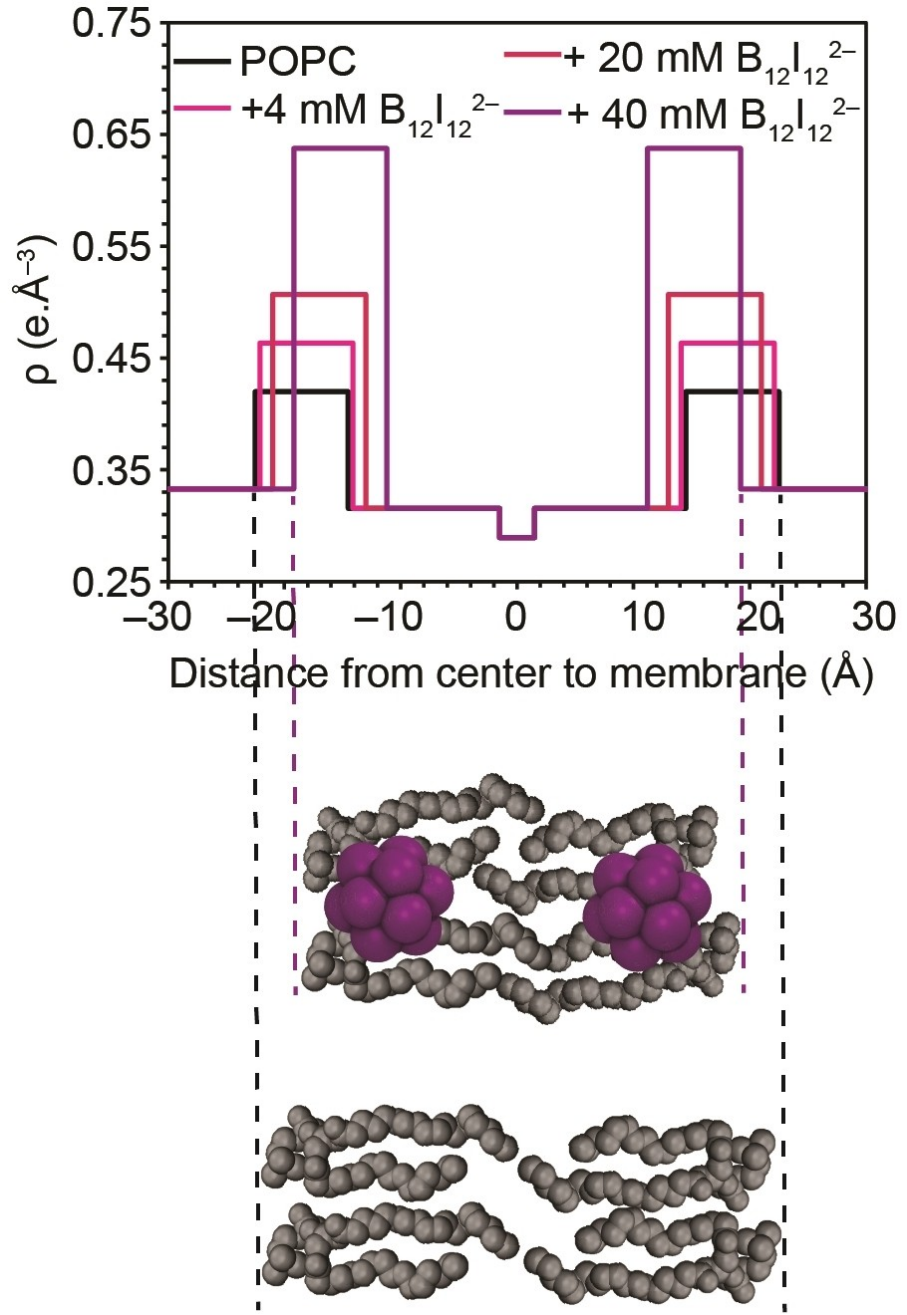
Electron density profile (top) of POPC alone (20 mM, black line) and in the presence of B_12_I_12_^2−^ (4 mM: pink line, 10 mM: dark pink, 20 mM: purple) and (bottom) illustration of the presumed cluster position in the membrane.

To obtain experimental data on the precise transversal localization of the cluster anions within the lipid bilayer and on the membrane thickness, we performed SAXS experiments of POPC multilamellar vesicles in the absence and presence of B_12_I_12_^2−^. To achieve sufficient scattering intensity and low noise over signal ratio in the SAXS experiments, we worked (*i*) at high lipid concentrations (20 mM), providing multilamellar liposomes, and with (*ii*) B_12_I_12_^2−^ which has the highest electron density among the dodecaborate clusters.^[89]^ The experimental raw data are shown in Figure S19, from which the electron‐density profile across the lipid bilayer was modeled with a simple box model, see Figure 5.

In the absence of B_12_I_12_^2−^, neat POPC liposomes showed the classical electron density profile with a total bilayer thickness of 45 Å, in agreement with literature,^[90]^ with a maximum electron density in the region of the polar head groups and a minimum electron density in the region of the terminal methyl group of the alkyl chains. Upon B_12_I_12_^2−^ addition (up to 40 mM), the electron density in the polar head region increased steeply, indicating a strong interaction between the cluster anions and the liposomes and confirming the localization of the cluster in the polar head region. The strong increase in electron density in the polar head (Figure 5) was used to estimate the B_12_I_12_^2−^/lipid molar ratio in the multilamellar vesicles upon increasing the B_12_I_12_^2−^ concentration (Figure S20), reaching values up to one cluster per 4–10 lipid molecules, again comparable to the approximate stoichiometries in the other lipid assemblies (monolayers and lipid bilayers, see above). Moreover, in Figure 5, the high electron density region continuously shifted toward the center of the bilayer upon addition of the cluster. This suggests that B_12_I_12_^2−^ binding causes a significant decrease in the bilayer thickness from 45 down to 38 Å (for 1 : 1 cluster/lipid ratio), a thinning phenomenon that has been reported for inclusion of certain organic molecules[^90^, ^91^, ^92^] into the polar headgroup region. SAXS measurements also pointed to a partial disruption of the multilamellar structure of the vesicles (peeling of the liposome structure) through the disappearance of the smectic scattering peaks (Figure S19); this is in line with the high affinity and incipient membrane lytic propensity of the heaviest and largest boron cluster anion. Our MD simulations confirmed the transversal positioning of the cluster ions (see above and Figure 4b) as well as the membrane thinning (Figure S17 and S18).

### Mechanistic Implications

Superchaotropic anions such as dodecaborates are unique in that they are both highly hydrophilic as well as lipophilic. They display a distinct affinity to hydrophobic cavities and interfaces,[^12^, ^13^] but in contrast to hydrophobic and amphiphilic molecules, they are highly water soluble, without a pronounced self‐assembly tendency to form microheterogeneous dispersions. In fact, structurally, they lack the classical head‐and‐tail characteristics required for amphiphiles.[^93^, ^94^] Thermochemically, the interactions between chaotropes and organic matter are enthalpically driven,^[13]^ as opposed to the entropically driven hydrophobic or amphiphilic aggregation. The high membrane affinity and lipid solubility of borate clusters observed in our present study provide an exceptional manifestation of their lipophilicity.

**Figure 6:**
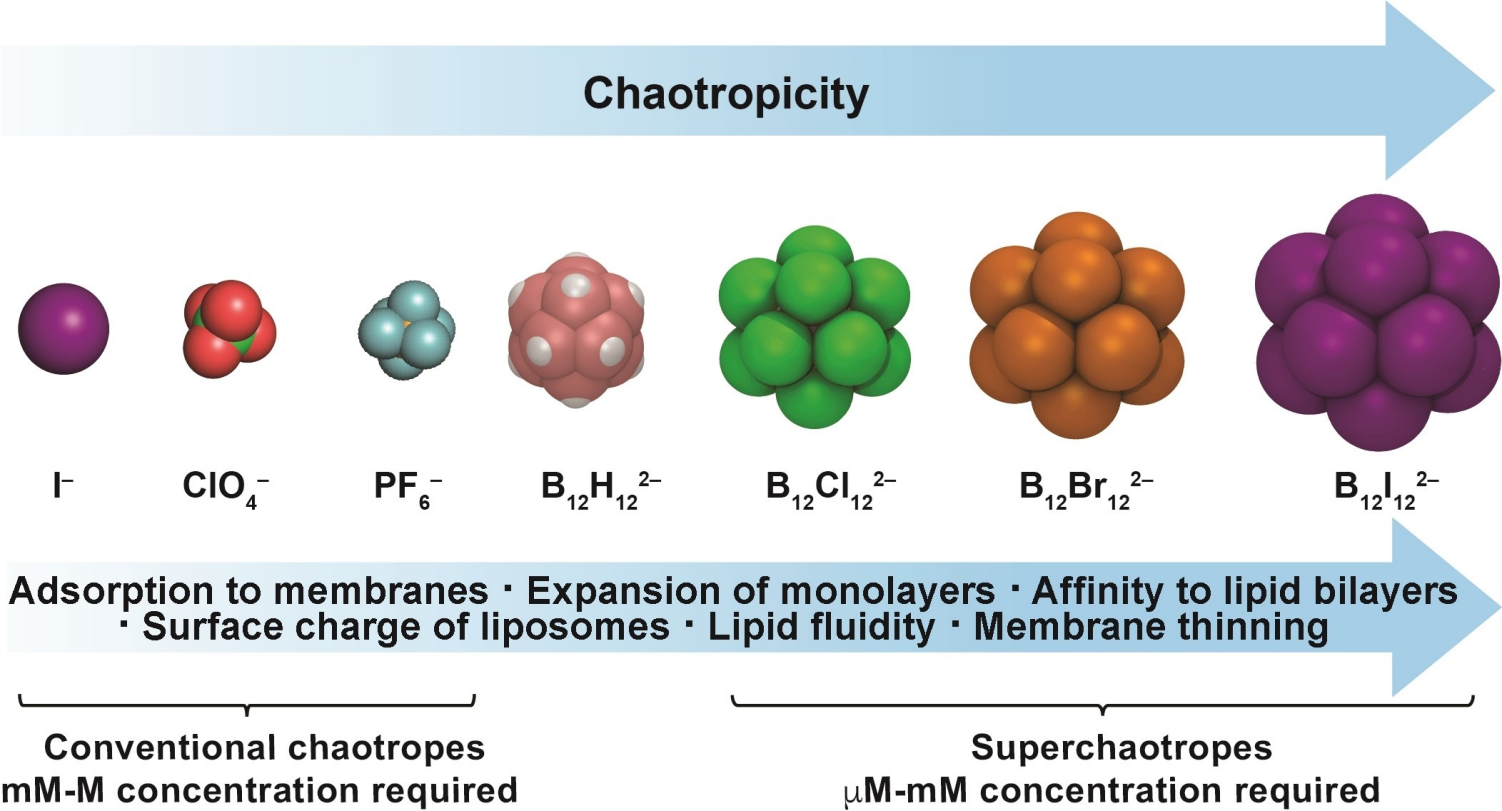
Effects of anions on lipid membranes in dependence on their chaotropicity and the required concentrations to produce significant effects.

**Figure 7:**
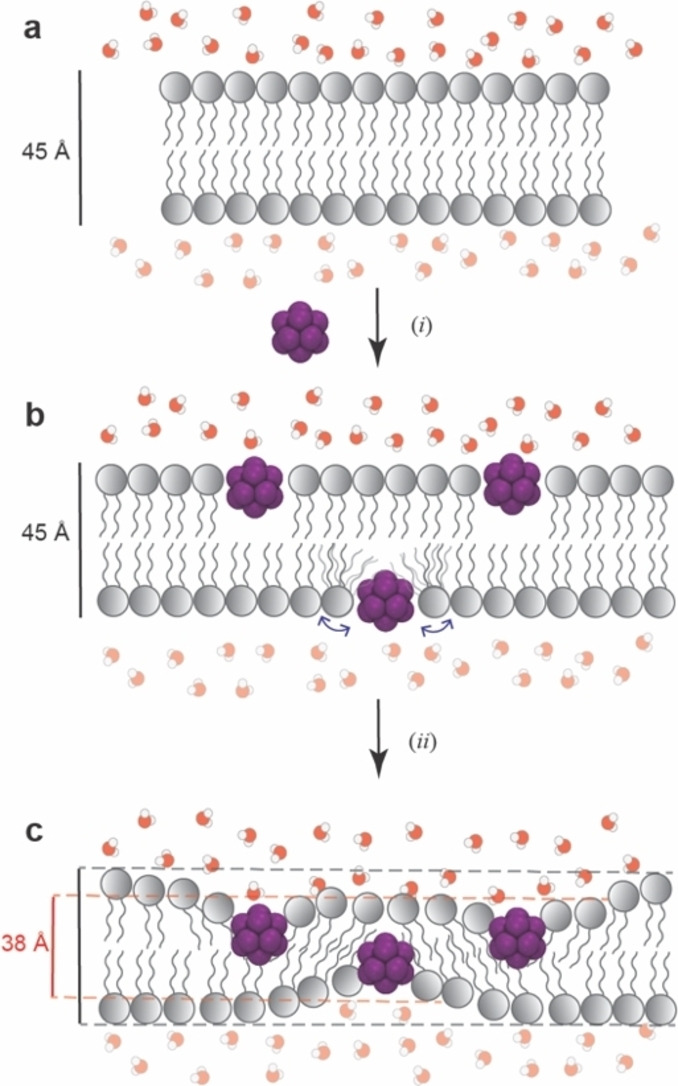
Proposed mechanism of boron cluster insertion. **a**) Unperturbed lipid bilayer. **b**) Cluster insertion pushes apart the polar heads, subsequently the hydrocarbon chains reorient, stimulating **c**) membrane fluidity and thinning. Note that **b**) is a hypothetical state, only drawn for visualization as a stepwise process, affecting (*i*) expansion and (*ii*) thinning.

The performed monolayer and bilayer measurements point to (*i*) an adsorption of boron clusters to POPC and DPPC lipid bilayers in their liquid phase, (*ii*) an expansion, (*iii*) an increase in fluidity, and (*iv*) a membrane thinning, with an increasing tendency for the larger halogenated ones. The MD simulations are in full qualitative conformity with these experimental results, as summarized in Figure 6. The combined experimental and theoretical results allow us to advance a mechanistic rationalization of all effects at an atomistic level, which is shown in Figure 7. Accordingly, the boron clusters immerse into the headgroup regions of the lipid layer. As the size of the clusters is smaller than the length of the lipids, such a full immersion into the headgroup region would result in void space below the clusters (Figure 7b). Invariably, this void space will be filled by alkyl chains protruding sideways below the boron clusters and will eventually produce a local thinning of the bilayer (Figure 7c). This sequence of supramolecular events will result necessarily in a lipid expansion (as observed by Langmuir trough experiments) as well as a thinning of the membrane (as observed by SAXS) since the lipids would have to realign. This translates into an increased membrane fluidity (as observed by Langmuir trough experiments) as well as a decreased phase‐transition temperature (as observed by DSC for DPPC, Table 2, Figure S15a). Lastly, because the adsorption process is driven by the chaotropic effect, the absolute magnitude of all experimental effects grows with the affinity of the boron clusters (as revealed by ITC), which increases with cluster size. Notably, the effects of the boron clusters are stronger than those for conventional chaotropes and require about 3 orders of magnitude lower concentrations (Figure 6). The largest cluster, B_12_I_12_^2−^, showed also incipient membrane‐lytic effects, which may account for the previously observed hemolytic effect^[89]^ and its cellular toxicity.[^29^, ^32^]

## Conclusions

Boron clusters are a privileged family of inorganic anions with superchaotropic character.[^12^, ^13^] Due to their unique properties, they have been applied in numerous fields,[^6^, ^7^, ^8^, ^9^, ^10^, ^11^] mostly in medicinal chemistry,[^16^, ^17^] because of their degradation resistance in biological environments.^[15]^ Due to their medical and biological relevance, we have comprehensively studied the intermolecular interactions of dodecaborate cluster anions on lipidic membranes with a portfolio of experimental techniques and MD simulations. The combined experiments showed strong adsorption of the halogenated boron cluster anions to the polar headgroup region of zwitterionic lipid membranes, which results in the manifold of effects in Figure 6. They can truly be referred to as “superchaotropic” anions, because their effects on membranes exceed the effects documented for regular chaotropic anions by far. In particular, the clusters unfold their biophysical effects already at μM concentrations, while conventional chaotropes require frequently M concentrations,[^60^, ^61^, ^62^, ^65^, ^67^, ^75^, ^76^, ^95^] at which general salt and osmotic effects greatly limit their biocompatibility. These assets could expand the widespread application of the chaotropic effect for the resolution of membranes and the isolation of membrane‐bound proteins.^[96]^ The disclosed cluster‐membrane interactions will be relevant for their recently observed membrane transport activity,[^29^, ^30^, ^53^] and for the design of improved bioactive cluster derivatives. Lastly, the results should be transferable to other boron cluster anions such as the recently studied percyano derivative[^97^, ^98^] as well as hydrolytically stable polyoxometalates.[^53^, ^55^, ^99^, ^100^]

## Conflict of Interests

The authors declare no conflict of interest.

1

## Supporting information

As a service to our authors and readers, this journal provides supporting information supplied by the authors. Such materials are peer reviewed and may be re‐organized for online delivery, but are not copy‐edited or typeset. Technical support issues arising from supporting information (other than missing files) should be addressed to the authors.

Supporting Information

## Data Availability

The data that support the findings of this study are available in the supplementary material of this article.
