# Supplementary material for: Boron Cluster Anions Dissolve En Masse in Lipids Causing Membrane Expansion and Thinning
Source: Angew Chem Int Ed Engl. 2024 Oct 31;63(51):e202412834. doi: 10.1002/anie.202412834 (PMC11627137; doi:10.1002/anie.202412834)
Supplement: Supplementary file 1 — Supporting Information [file ANIE-63-e202412834-s001.pdf]

Angewandte Chemie
Eine Zeitschrift der Gesellschaft Deutscher Chemiker

Supporting Information

Boron Cluster Anions Dissolve *En Masse* in Lipids Causing Membrane Expansion and Thinning

A. Barba-Bon, A. El Haitami, C. Pasquier, I. Nikšić-Franjić, O. Diat, P. Bauduin, S. Cantin, W. M. Nau**

Supporting Information

Boron Cluster Anions Dissolve *en masse* in Lipids Causing Membrane Expansion and Thinning

Andrea Barba-Bon*[a] Alae El Haitami,[b] Coralie Pasquier,[c] Ivana Nikšić-Franjić,[a] Olivier Diat,[c] Pierre Bauduin,[c] Sophie Cantin,[b] and Werner M. Nau*[a]

[a] Dr. Andrea Barba-Bon, Dr. Ivana Nikšić-Franjić, Prof. Dr. Werner M. Nau
School of Science, Constructor University
Campus Ring 1, D-28759 Bremen, Germany
E-mail: abarbabon@constructor.university; wnau@constructor.university

[b] Dr. Alae El Haitami, Prof. Dr. Sophie Cantin
Laboratoire de Physicochimie des Polymères et des Interfaces (LPPI)
Université de Cergy-Pontoise
5 mail Gay Lussac, F-95031Cergy-Pontoise-Cedex, France.

[c] Dr. Coralie Pasquier, Prof. Dr. Pierre Bauduin, Prof. Dr. Olivier Diat
Institut de Chimie Séparative de Marcoule, CNRS UMR 5257,
CEA, Université de Marcoule, ENSCM,
F-30207 Bagnols sur Cèze Cedex, France.

Table of Contents

1. *Materials and Methods* 2
2. *Phases in the Lipid Monolayer* 4
3. *Phases in the Lipid Bilayer* 4
4. *Langmuir Monolayer Measurements* 5
5. *Brewster Angle Microscopy* 6
6. *Dynamic Light Scattering* 7
7. *ζ-Potential Measurements* 8
8. *Isothermal Titration Calorimetry* 9
9. *Differential Scanning Calorimetry* 13
10. *Molecular Dynamics Simulations* 14
11. *Small-angle X-ray Scattering* 15
12. *References* 18

SUPPORTING INFORMATION

1. Materials and Methods

Materials

1-palmitoyl-2-oleoyl-*sn*-glycerol-3-phosphocholine (POPC), 1,2-dipalmitoyl-2-*sn*-3-phosphocholine (DPPC), and 1,2-dipalmitoyl-*sn*-glycero-3-phosphorylglycerol (DPPG), were obtained from Sigma-Aldrich (Germany) and used without further purification. Boron clusters were acquired as sodium salts from Katchem (Czech Republic).

Langmuir monolayer experiments

The surface pressure (π) - area (*A*) isotherms were measured at the air-water interface with a Kibron (µTroughX) Langmuir-Blodgett through equipped with two compression barriers made of Teflon. The surface pressure was monitored during film compression with a hydrophilic metal oxide alloy probe, which was cleaned before use with a Bunsen burner. All experiments were conducted at 20±1°C, the temperature in the subphase was kept constant with a recirculating thermostat. The through was filled with ultra-pure water or the boron cluster solution as subphase. Phospholipid monolayers were formed by spreading 20 µL of a 1 mg/mL chloroform solution of the lipid (0.45 mg/mL in chloroform/ethanol 9/1 v/v for DPPG) with a Hamilton syringe, and 10 mins were allowed to ensure solvent evaporation prior the compression. Lipid stock solutions were stored in the dark at 4°C. A constant compression speed of 25.5 mm/min was applied. All isotherms were reproduced at least twice.

Liposome preparation

A thin lipid film was prepared by evaporating a lipid solution with a stream of nitrogen and dried in vacuo overnight (500 µL of a 25 mg/mL chloroform stock solution for POPC and DPPC, and 1 mL of 5 mg/mL in chloroform/ethanol 5/1 v/v for DPPG). To prepare the liposomes, the lipid film was rehydrated (for 30 min at ambient temperature for POPC and for 40 min at 55°C for DPPC and DPPG) with 1 mL 10 mM Tris, pH 7,4, subjected to 7 freeze/thaw cycles, and 15 extrusions through a polycarbonate membrane (pore size 100 nm; ambient temperature for POPC and 55°C for DPPC and DPPG). The vesicle size was confirmed by DLS and the phospholipid concentration was determined by Stewart assay.[1]

Dynamic Light Scattering (DLS)

DLS experiments were carried out on a Malvern Instruments DTS Nano 2000 Zeta-Sizer. Liposomes (250 µM phospholipid) were incubated with different concentrations of cluster for 2 hours at 25ºC. All experiments were conducted with 10 mM Tris buffer pH 7,4. Measurements were recorded in a 1,5 mL disposable cuvette, using a 90º scattering optics at 25ºC; twelve individual determinations were averaged.

ζ-Potential

Zeta potential (ζ) values were measured on a Malvern Instruments DTS Nano 2000 Zeta-Sizer. Liposomes (250 µM phospholipid) were incubated with different concentrations of the clusters for 2 hours at 25 or 50°C. All experiments were conducted with 10 mM Tris buffer, pH 7.4. Measurements were recorded in disposable folded capillary cells (DTS1070) and evaluated based on the Smoluchowski equation (particle diameter ~120 nm >> Debye length ~ 1 nm); fifteen individual determinations were averaged.

Isothermal Titration Calorimetry (ITC)

All experiments were performed in a VP-ITC MicroCalorimeter from MicroCal, Int., at atmospheric pressure and 25°C. Solutions were degassed and thermostated prior to the titration experiments in a ThermoVac accessory. A constant volume of dodecaborate cluster (10 µL per injection) was injected into the liposomal suspension in buffer (10 mM Tris, pH 7.4) to determine the cluster-lipid apparent binding affinity. Dilution heats were determined by titration of cluster into buffer and subtracted from the reaction heat. The neat reaction heat was fitted with Origin 7.0 software by using a one-set-of-sites model to obtain the complex stability constant (K_a) and molar reaction enthalpy ($\Delta H°$). The free energy ($\Delta G°$) and entropy changes ($\Delta S°$) were obtained according to $\Delta G° = -RT\ln K_a = \Delta H° - T\Delta S°$.

Differential Scanning Calorimetry (DSC)

The liposomal phase transitions were measured on a VP-DSC MicroCalorimeter from MicroCal, Int. All experiments were conducted with 10 mM Tris buffer, pH 7.4, which was loaded in the reference cell. Liposomes were incubated with different concentrations of the clusters for 2 hours at ambient temperature. Prior to injection, both liposomes (with and without cluster) and buffer (reference) were degassed and thermostated. Up-scans were performed at a scan rate of 90°C/h from 20-60°C (or 90°C for DPPG), while down-scans ran at a scan rate of 60°C/h. Six scans

were collected with a filtering time of 2 s and 15 min equilibration time. The background with buffer (in both cells) was subtracted. Curve fitting was performed using Origin 5.0 software from MicroCal by using the Levenberg-Marquardt non-linear least-square method and a non-two-state model ("model 2") to obtain the phase transition temperature (T_m), the calorimetric enthalpy (ΔH), and the van't Hoff enthalpy (ΔH_{vH}). In the non-two-state model, the calorimetric enthalpy is determined only by the area under the transition peak while the van't Hoff enthalpy is determined only by the shape of the transition. All transitions were reproduced at least twice.

Note that the phase transition in lipid bilayers, unlike the crystal to liquid transition in organic compounds, comprises more than an initial and final state. Intermediate states (coexistence between fluid sections in the gel phase) usually form during the transition and a “non-two-state” model is needed in these cases to analyze the melting process, providing both calorimetric enthalpy (ΔH_t) and van't Hoff enthalpy (ΔH_{vH}). When $\Delta H_t \neq \Delta H_{vH}$ in a transition, it is considered as “cooperative”, the ratio $(\Delta H_t / \Delta H_{vH})^2$ is the cooperativity parameter (σ) and the degree of cooperativity between the lipid molecules is quantified by the cooperative unit C.U. = $1/\sqrt{\sigma}$,[2-3] which is the number of lipids per interfacial lipid within a domain at the center of the transition.[4]

Molecular dynamics simulations

Classical molecular dynamics (MD) simulations were employed to model the interactions of POPC lipids with dodecaborate clusters in water. A model POPC lipid bilayer was generated by using the CHARMM-GUI Membrane Builder module graphical interface[5] and distributing POPC molecules in a 90 $\times$ 65 Å water-box, resulting in a bilayer involving two leaflets of 64 lipids each. Clusters were positioned outside the membrane (5 above and 5 below the leaflets, 10 clusters in total) and solvated with explicit TIP3P (*Transferable Intermolecular Potential with 3 Points*)[6] water molecules; Na+ counterions were added to neutralize the system. The Lipid21 force field[7] was employed for the lipids, and the boron clusters were reparametrized by using the VFFDT (*Visual Force Field Derivation Toolkit*) software[8] that uses the Seminario method to obtain the missing bond, angle, and non-bonding parameters. VFFDT has previously shown good performance for obtaining the force-field parameters of boron compound in molecular dynamics simulations.[9-10] The harmonic vibrational frequencies necessary for angle and bond parameters were calculated at the B3LYP/6-31G(d,p) level of theory[11-12] and atomic partial charges according to the RESP (*Restrained Electrostatic Potential Charges*) procedure[13] (HF/6-31G(d)). For quantum-chemical calculations the Gaussian 16 program package, Revision C.01,[14] was used. Each simulation was performed in four steps: minimization, heating, equilibration, and final production (MD simulation) of the system. The geometries were optimized in the first 5000 steps with the steepest descent method and in the remaining steps with the conjugate gradient method without any constraints. After minimization, the system was heated for 5 ps from 0-100 K at constant volume (*NVT* ensemble, Langevin thermostat) and 100 ps from 100-303 K at constant pressure (*NpT* ensemble, Monte Carlo barostat, anisotropic pressure coupling, pressure relaxation time 2 ps). An equilibration step of 100 ns was performed at constant pressure, while the production step was obtained for 500 ns. Three-dimensional periodic boundary conditions were used throughout. The time steps for all simulations were 2 fs and the coordinates were saved. The cutoff for short-range Coulomb interactions and van der Waals interactions was set to 12 Å, with the switching function for the latter turned on beyond 10 Å. Long-range interactions were handled by using the particle-mesh Ewald procedure (PME).[15] Bonds involving hydrogens were constrained with SHAKE.[16] Simulations were performed in the Amber22 program package,[17-18] and data analysis as well as visualization were done in the Visual Molecular Dynamics (VMD)[19] and Pymol[20] software packages.

The electron density profiles and the lipid-order parameters were obtained by using the AMBER trajectory analysis module cpptraj from the last 100 ns of MD production step.[7,17-18] The lipid-order parameter, S_{CD}, was calculated for all carbon atoms of the *sn*-palmitoyl and *sn*-oleoyl chains from the simulated bilayers[21] as $S_{i(\mathrm{CH})} = \langle 3\cos^2\theta_{i(\mathrm{CH})} - 1\rangle/2$, where $\theta_{i(\mathrm{CH})}$ is the angle between the bilayer normal (which typically coincides with the z-axis) and the methylene C–H bonds of the carbons *i* in the selected POPC chain. The angular brackets indicate both temporal and molecular averages.

Small-angle X-ray Scattering (SAXS)

SAXS measurements, using Mo radiation (λMo = 0.071 nm), were performed on a bench built by XENOCS. The scattered beam was recorded by using a large online scanner detector (diameter: 345 mm, from MAR Research). A large q-range (0.2 to 40 nm^{-1}) was covered with an off-center detection. The collimation was applied with a 12:∝ multilayer Xenocs mirror (for Mo radiation) coupled to two sets of scatterless FORVIS slits providing a 0.8 × 0.8 mm X-ray beam at the sample position. Pre-analysis of data was performed in PySAXS software. 2-mm quartz capillaries were used as sample containers. Usual corrections for background (empty cell and detector noise) subtractions and intensity normalization with a high-density polyethylene film as a standard were applied. Experimental resolution was $\Delta Q/Q$ = 0.05. Silver behenate in a sealed capillary was used as the scattering vector calibration standard.

2. Phases in the Lipid Monolayer

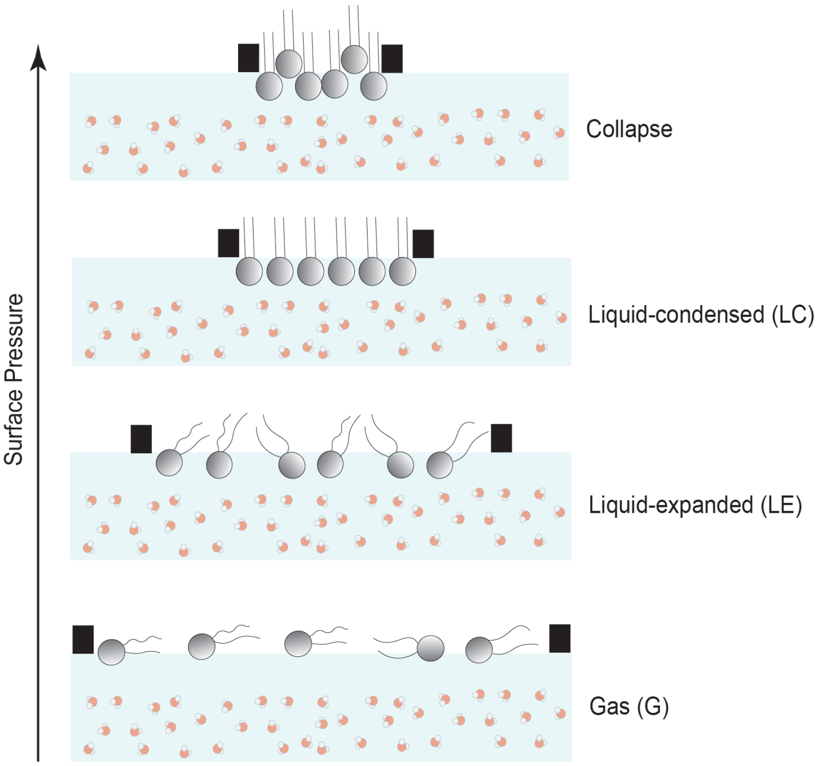

Scheme S1: Illustration of the different phases in a lipid monolayer. Molecules in the gas phase are loosely packed at the water surface and behave like a 2D gas. In the LE phase, molecules behave like a 2D liquid and in the LC phase, molecules are in a 2D semicrystalline phase. Further compression results in film collapse, where an inflection appears in the isotherm.

3. Phases in the Lipid Bilayer

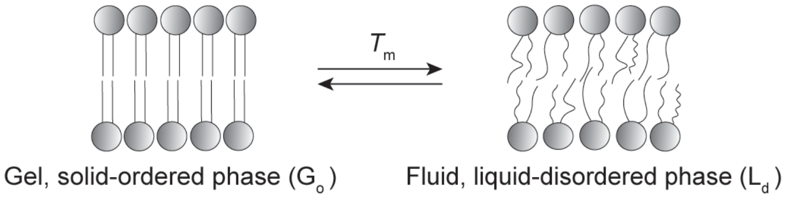

Scheme S2: Illustration of the different phases in a lipid bilayer. In the gel phase, the acyl side chains of the lipids are well packed, resulting in a higher rigidity leading to a low mobility. Heating above their phase transition will “melt” the lipids to form the liquid-disordered phase, which is more fluid.

4. Langmuir Monolayer Measurements

POPC monolayer

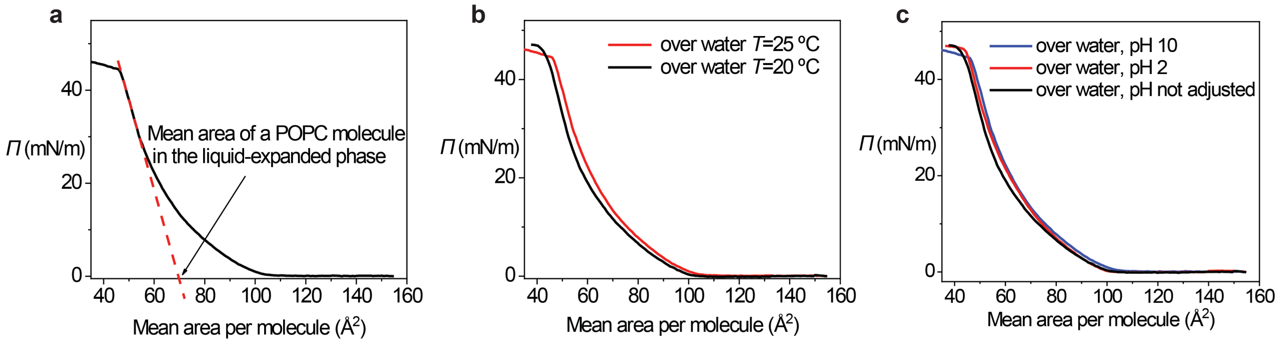

Figure S1: π-A isotherms for POPC monolayers on a neat water subphase. **a** Mean area is calculated by extrapolation of the sharpest linear region to the x-axis. **b** Temperature and **c** pH effect on the POPC monolayer. The surface area of POPC over neat water changes only slightly in dependence on ambient temperature or pH.

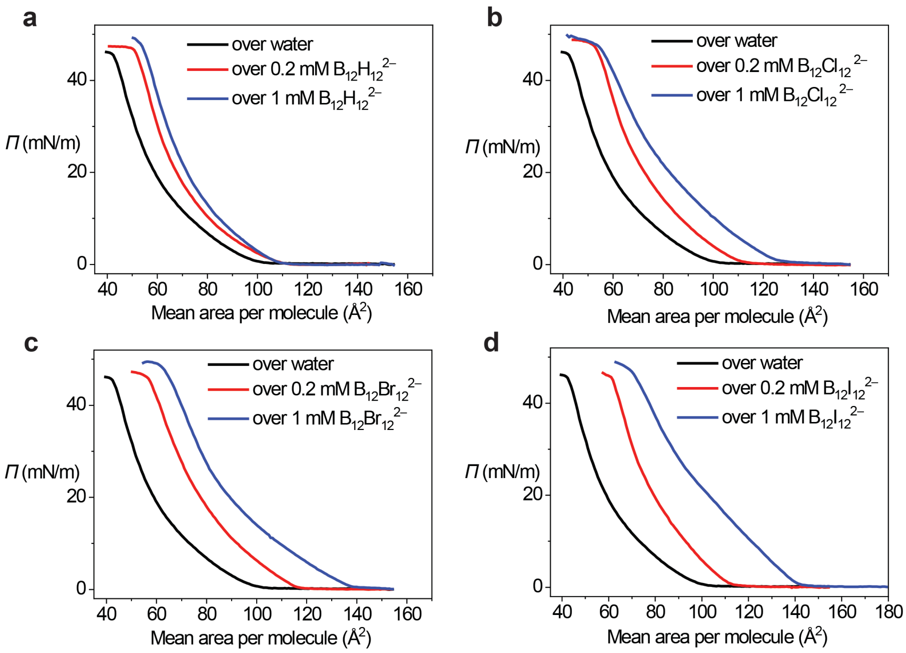

Figure S2: Interaction between different boron clusters and the zwitterionic POPC monolayer. π-A isotherms for POPC monolayers on a subphase with **a** $B_{12}H_{12}^{2-}$, **b** $B_{12}Cl_{12}^{2-}$, **c** $B_{12}Br_{12}^{2-}$, and **d** $B_{12}I_{12}^{2-}$ at two different concentrations. Higher cluster concentrations yielded larger surface areas.

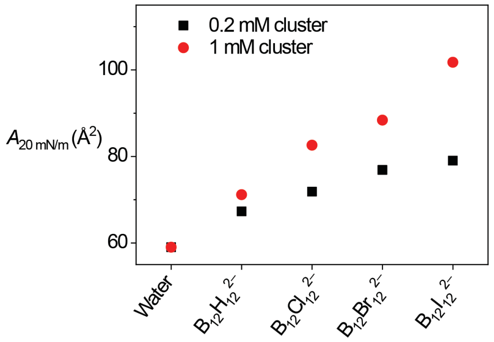

Figure S3: Mean area per molecule at a constant surface pressure of 20 mN/m in the absence and presence of boron clusters (0.2 and 1.0 mM), ordered according to increasing chaotropicity, from left to right.

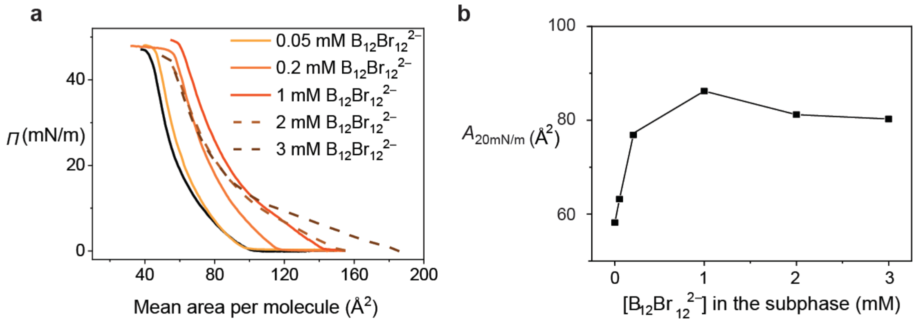

Figure S4. a π-A isotherms for POPC monolayers on a subphase containing increasing $B_{12}Br_{12}^{2-}$ concentrations. **b** Mean area per molecule at a constant surface pressure of 20 mN/m as a function of $B_{12}Br_{12}^{2-}$ concentration. Increasing cluster concentration produced an expansion to larger areas (up to 99 Å^2 in the presence of 1 mM cluster); higher $B_{12}Br_{12}^{2-}$ concentrations yielded reduced expansions (88 Å^2 $B_{12}Br_{12}^{2-}$ in presence of 3 mM). However, the lift-off (A_1, first increase in subphase pressure on compression) was larger at higher cluster concentrations, pointing to a stronger cluster adsorption. The apparent "compression" at higher $B_{12}Br_{12}^{2-}$ concentrations could be explained as a possible misreading of the sensor due to the increased rigidity of the monolayer in the presence of the cluster (see BAM images, Fig.S5, for details).

5. Brewster Angle Microscopy

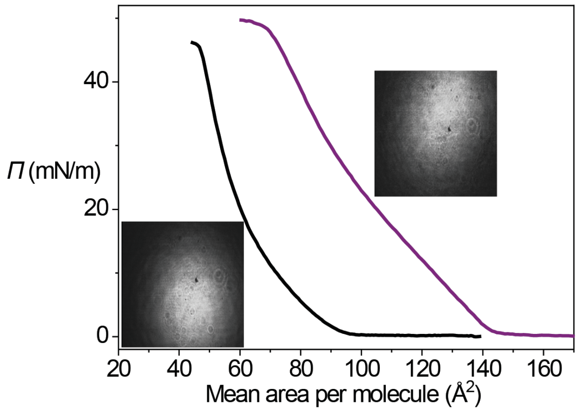

Figure S5. π-A isotherms for POPC monolayers over a water (black line) *vs.* a $B_{12}I_{12}^{2-}$ (1.0 mM, purple line) subphase (1 mM) with BAM images for the selected LE phase (from 1 to 45 mN/m). Experiments were conducted with a POPC monolayer on neat water and $B_{12}I_{12}^{2-}$ in the subphase (the most chaotropic cluster) and the goal was to visualize the POPC phases during monolayer compression. In both cases, from 1 to 45 mN/m only a single phase (LE) was detected[22] but the monolayer was found to be more rigid in the presence of $B_{12}I_{12}^{2-}$. Image sizes: 600*600 μm^2.

6. Dynamic Light Scattering

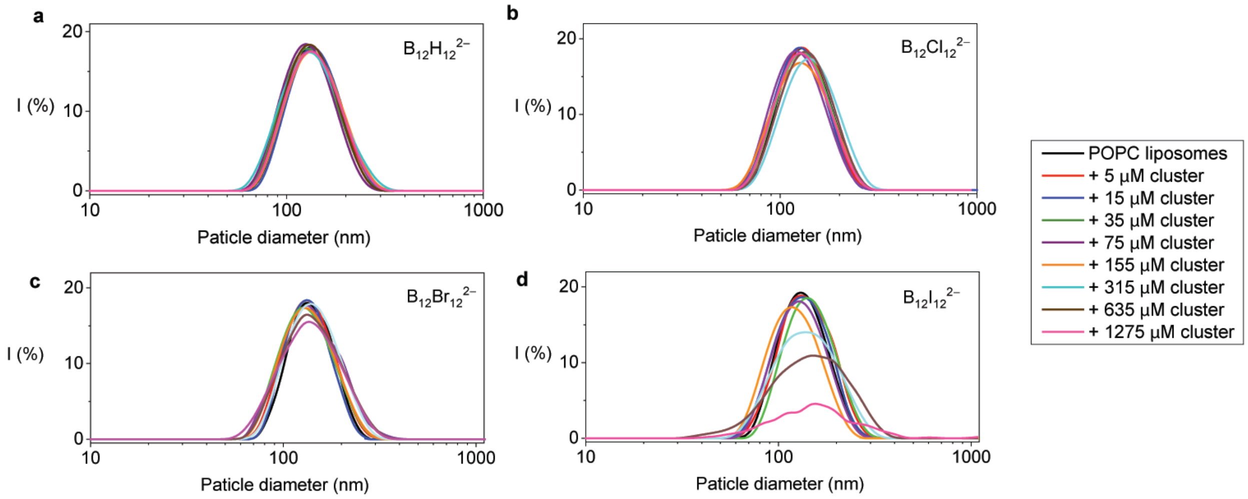

Figure S6: Size distribution (by DLS) of POPC liposomes (250 µM phospholipid in 10 mM, Tris pH 7.4) before (black line) and after addition of increasing concentrations of **a**. $B_{12}H_{12}^{2-}$, **b**. $B_{12}Cl_{12}^{2-}$, **c**. $B_{12}Br_{12}^{2-}$, and **d**. $B_{12}I_{12}^{2-}$. Note the membrane lysis for $B_{12}I_{12}^{2-}$ above 300 µM.

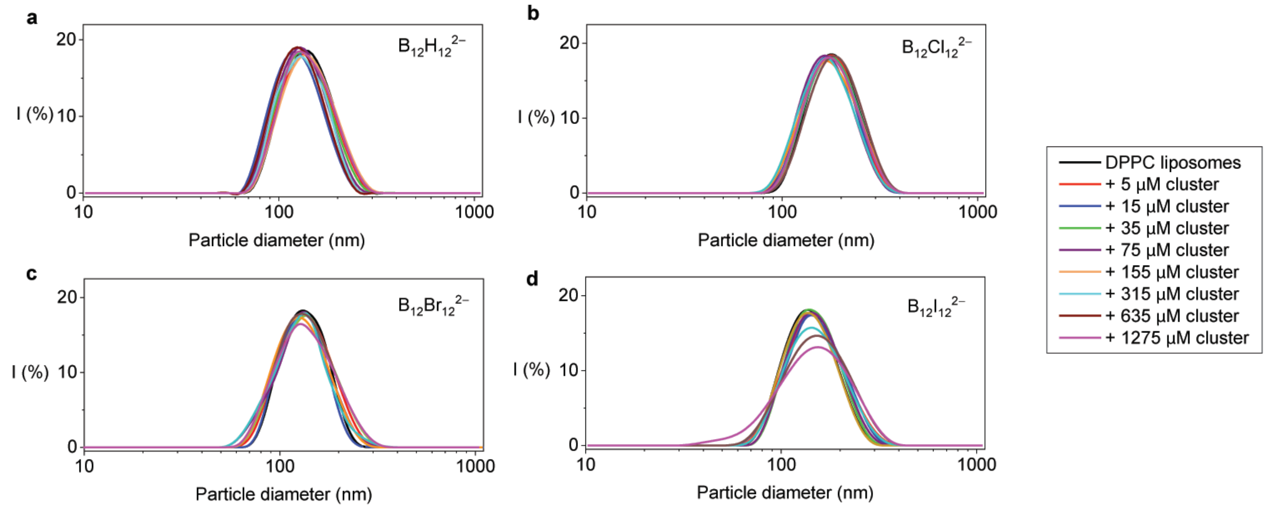

Figure S7: Size distribution (by DLS) of DPPC liposomes (250 µM phospholipid in 10 mM Tris, pH 7.4) before (black line) and after addition of increasing concentrations of **a** $B_{12}H_{12}^{2-}$, **b** $B_{12}Cl_{12}^{2-}$, **c** $B_{12}Br_{12}^{2-}$, and **d** $B_{12}I_{12}^{2-}$.

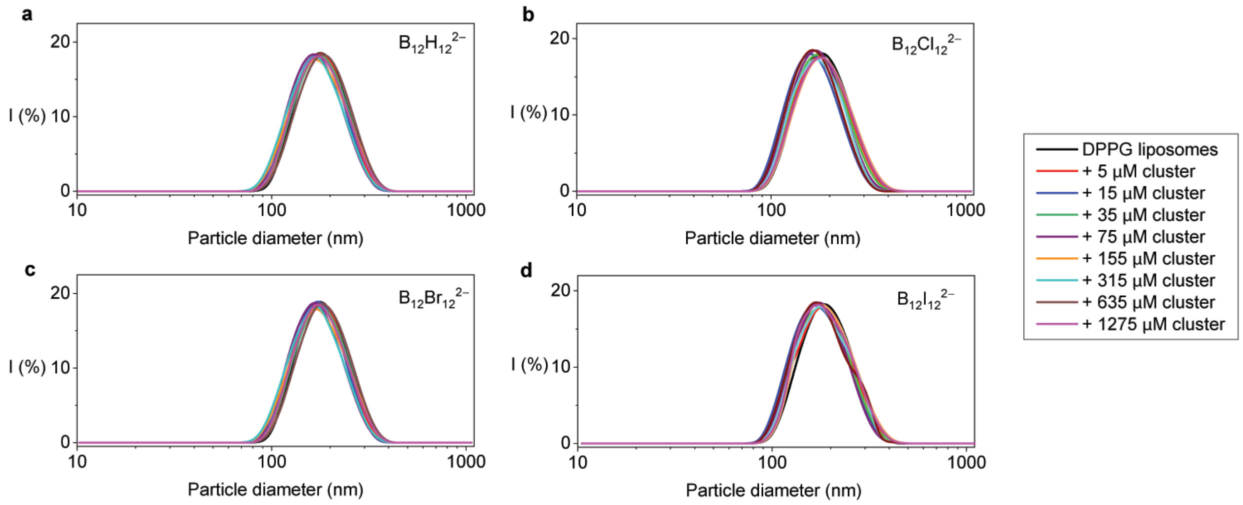

Figure S8: Size distribution (by DLS) of DPPG liposomes (250 µM phospholipid in 10 mM Tris, pH 7.4) before (black line) and after addition of increasing concentrations of **a** $B_{12}H_{12}^{2-}$, **b** $B_{12}Cl_{12}^{2-}$, **c** $B_{12}Br_{12}^{2-}$, and **d** $B_{12}I_{12}^{2-}$.

7. ζ-Potential Measurements

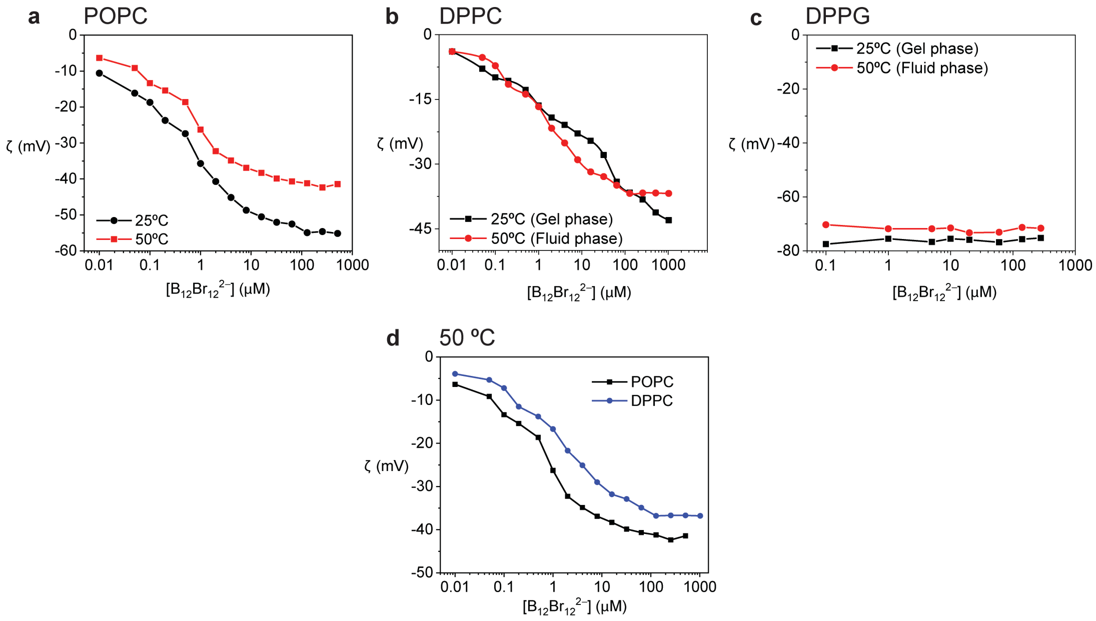

Figure S9: Zeta potential of **a** POPC, **b** DPPC, and **c** DPPG liposomes (250 μM phospholipid in 10 mM Tris, pH 7.4) as a function of $B_{12}Br_{12}^{2-}$ concentration, at 25ºC (black squares) and 50ºC (red dots). **d** Comparison of the effect of $B_{12}Br_{12}^{2-}$ in POPC (black square traces) and DPPC (blue dot traces), both at 50ºC. Liposomes were incubated with $B_{12}Br_{12}^{2-}$ for 2 h at the corresponding temperature.

8. Isothermal Titration Calorimetry

Table S1: Binding constants (K_a) and thermochemical data for the interaction of dodecaborate clusters with POPC, DPPC, or DPPG liposomes of different composition in buffer at 25 ºC.[a]

Boron cluster	Titration type [b]	POPC					DPPC	DPPG
		n	K_a / ($10^5 M^{-1}$)	ΔH/ (kcal/mol)	$T\Delta S$/ (kcal/mol)	ΔG/ (kcal/mol)		
$B_{12}H_{12}^{2-}$	direct	n.h.[c]	n.h.	n.h.	n.h.	n.h.		
	reverse	n.h.	n.h.	n.h.	n.h.	n.h.		
$B_{12}Cl_{12}^{2-}$	direct	10.4	4.1	–27.8	–20.2	–7.6		
	reverse	11.1[d]	1.2	–30.8	–23.9	–6.9		
$B_{12}Br_{12}^{2-}$	direct	7.3	5.3	–34.5	–26.6	–7.9	n.h.	n.h.
	reverse	7.7[d]	1.3	–36.8	–29.9	–6.9		
$B_{12}I_{12}^{2-}$	direct	6.5	6.2	–27.1	–19.1	–8.0		
	reverse	6.7 [d]	1.3	–32.8	–25.9	–6.9		

[a]Measured by ITC in 10 mM Tris buffer, pH 7.4; dodecaborate clusters used as sodium salts; error in data is 10% for the n values and K_a, and ± 0.5 kcal mol^{-1} for ΔH and $T\Delta S$, and ΔG (SD, duplicates). [b] Direct titration: liposome solution in the syringe added into cluster solution in the cell. Reverse titration: cluster solution in the syringe added into liposome solution in the cell. Dilution heats were determined by titration of cluster/liposome solution into buffer and subtracted from the reaction heats. [c] n.h.: No reaction heat was obtained. [d] $n = 1/n'$ where n' is the stoichiometric value from the reverse titration.

Table S2: Binding constants (K_a) and thermochemical data for the interaction of dodecaborate clusters with DPPC and DPPG liposomes in buffer at 55 ºC. [a]

Boron cluster	Titration type[b]	DPPC					DPPG
		n	K_a / ($10^5 M^{-1}$)	ΔH/ (kcal/mol)	$T\Delta S$/ (kcal/mol)	ΔG/ (kcal/mol)	
$B_{12}H_{12}^{2-}$	direct	n.h.[c]	n.h.	n.h.	n.h.	n.h.	
$B_{12}Cl_{12}^{2-}$	direct	7.9	0.7	–32.1	–24.7	–7.4	
$B_{12}Br_{12}^{2-}$	direct	7.4	2.1	–28.9	–20.9	–8.0	n.h.
$B_{12}I_{12}^{2-}$	direct	6.1	1.0	–28.2	–20.7	–7.5	

[a]Measured by ITC in 10 mM Tris pH 7.4; dodecaborate clusters used as sodium salts; error in data is 10% for the n values and K_a, and ± 0.5 kcal mol^{-1} for ΔH and $T\Delta S$, and ΔG (SD, duplicates). [b] Direct titration: liposome solution in the syringe added into cluster solution in the cell. Dilution heats were determined by titration of liposome solution into buffer and subtracted from the heats. [c] n.h.: No reaction heat was obtained.

POPC liposomes at 25 ºC in their liquid phase

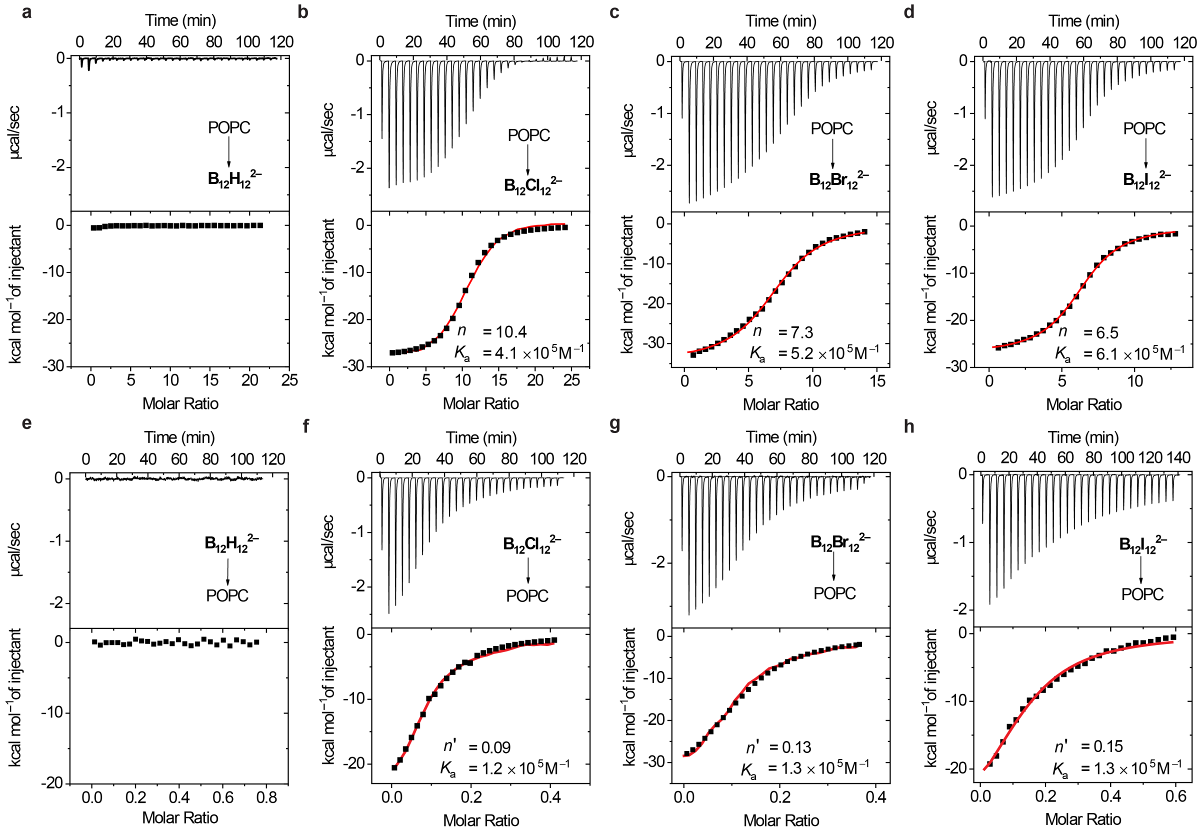

Figure S10: Microcalorimetric titrations in 10 mM Tris, pH 7.4 at 25 ºC. Thermograms (top panels) for the sequential injections of POPC liposome solutions into the boron cluster solutions (**a**, **b**, **c**, and **d**), or the reverse titrations where the boron cluster solutions were injected sequentially to the POPC liposome solutions (**e**, **f**, **g**, and **h**). The reaction heats were obtained from the integration of the calorimetric traces (bottom). Dilution heats were determined by the injections of boron clusters/POPC liposome solutions into buffer and subtracted from the reaction heat. Syringe/cell concentrations in mM: **a** 5.0/0.05; **b** 2.6/0.023; **c** 2.5/0.038; **d** 3/0.05. **e** 0.50/0.20; **f** 0.35/0.18; **g** 0.35/0.20; **h** 0.25/0.09.

DPPC liposomes at 25 ºC in their gel phase

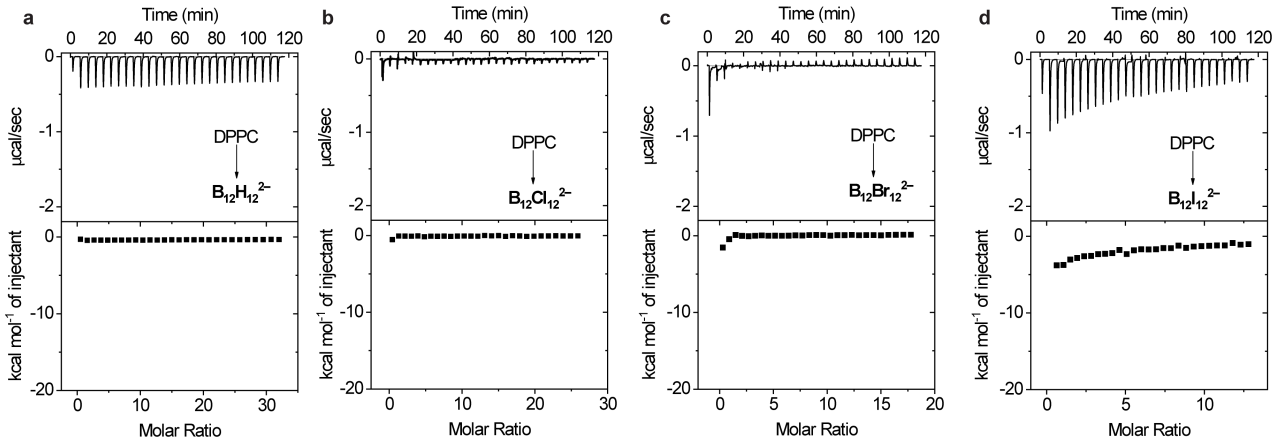

Figure S11: Microcalorimetric titration of DPPC liposomes with **a** $B_{12}H_{12}^{2-}$, **b** $B_{12}Cl_{12}^{2-}$, **c** $B_{12}Br_{12}^{2-}$, and **d** $B_{12}I_{12}^{2-}$. Raw ITC data (top) for the sequential injections of the boron clusters solutions to the liposome solutions. The apparent reaction heats were obtained from the integration of the calorimetric traces (bottom). All experiments were conducted in 10 mM Tris, pH 7.4 at 25ºC. Liposome/cluster solutions concentrations in mM: **a** 6.0/0.04; **b** 3.0/0.02; **c** 2.5/0.03; **d** 3/0.05.

DPPC liposomes at 55 ºC in their fluid phase

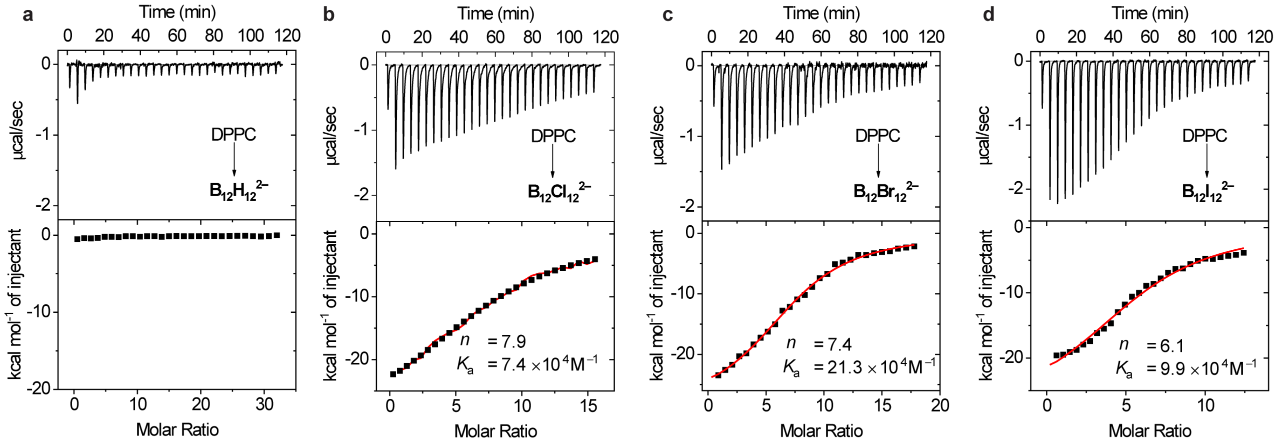

Figure S12: Microcalorimetric titrations in 10 mM Tris, pH 7.4 at 55 ºC. Thermograms (top panels) for the sequential injections of DPPC liposome solutions into the cluster solutions (**a**, **b**, **c**, and **d**). The reaction heats were obtained from the integration of the calorimetric traces (bottom). Dilution heats were determined by the injections of DPPC liposome solutions into buffer and subtracted from the reaction heat. Liposome/cluster solutions concentrations in mM: **a** 6.0/0.04; **b** 3.0/0.04; **c** 2.5/0.03; **d** 3.0/0.05.

DPPG liposomes at 25 ºC in their gel phase

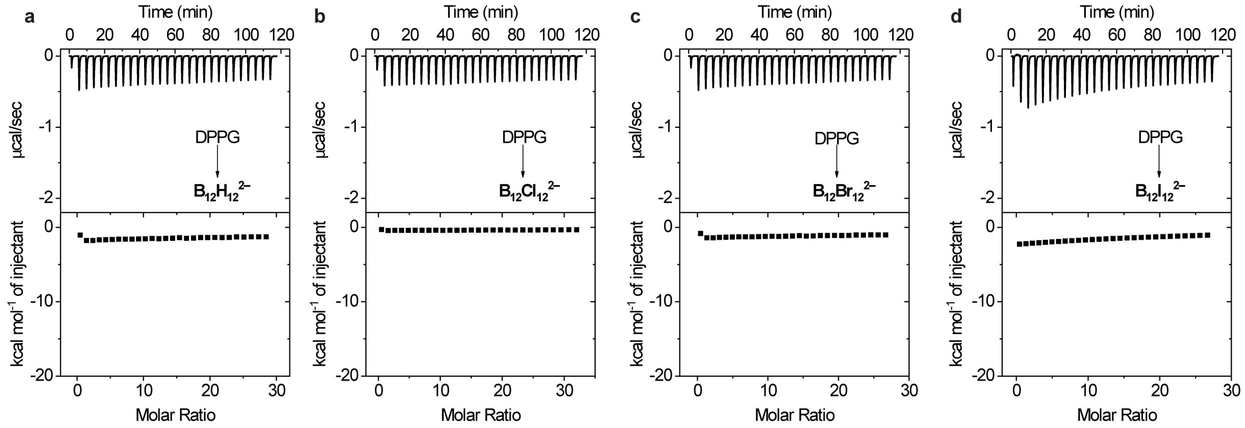

Figure S13: Microcalorimetric titration of DPPG liposome solutions with **a** $B_{12}H_{12}^{2-}$, **b** $B_{12}Cl_{12}^{2-}$, **c** $B_{12}Br_{12}^{2-}$, and **d** $B_{12}I_{12}^{2-}$. Raw ITC data (top) for the sequential injections of the boron cluster solutions to the liposome solutions. The apparent reaction heats were obtained from the integration of the calorimetric traces (bottom). All experiments were conducted in 10 mM Tris, pH 7.4. Liposome/cluster solutions concentrations in mM: **a** 4.0/0.03; **b** 3.0/0.04; **c** 2.5/0.02. **d** 2.5/0.02.

DPPG liposomes at 55 ºC in their fluid phase

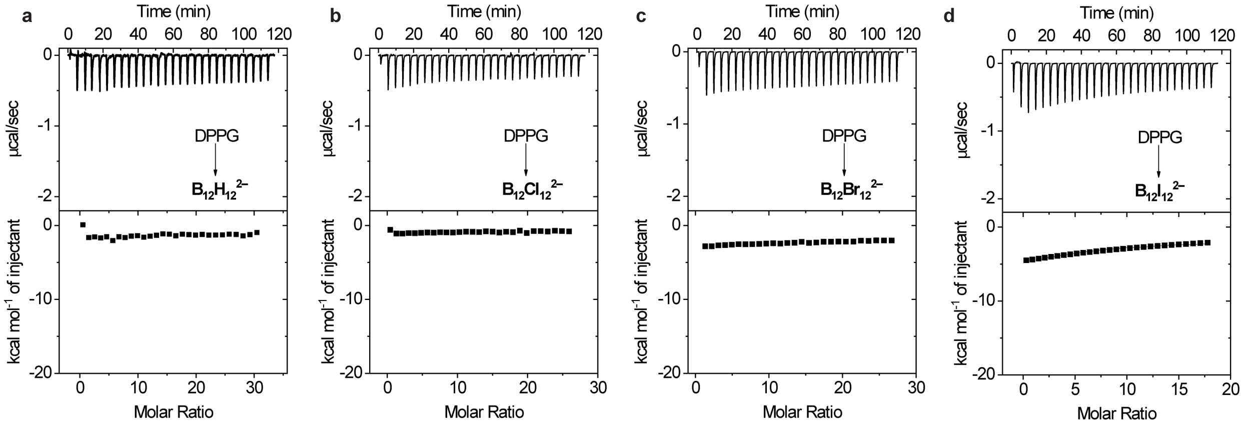

Figure S14: Microcalorimetric titration of DPPG liposome solutions with **a** $B_{12}H_{12}^{2-}$, **b** $B_{12}Cl_{12}^{2-}$, **c** $B_{12}Br_{12}^{2-}$, and **d** $B_{12}I_{12}^{2-}$. Raw ITC data (top) for the sequential injections of the boron cluster solutions to the liposome solutions. The apparent reaction heats were obtained from the integration of the calorimetric traces (bottom). All experiments were conducted in 10 mM Tris, pH 7.4. Liposome/cluster solutions concentrations in mM: **a** 3.5/0.03; **b** 2.5/0.02; **c** 2.5/0.02. **d** 2.5/0.03.

9. Differential Scanning Calorimetry

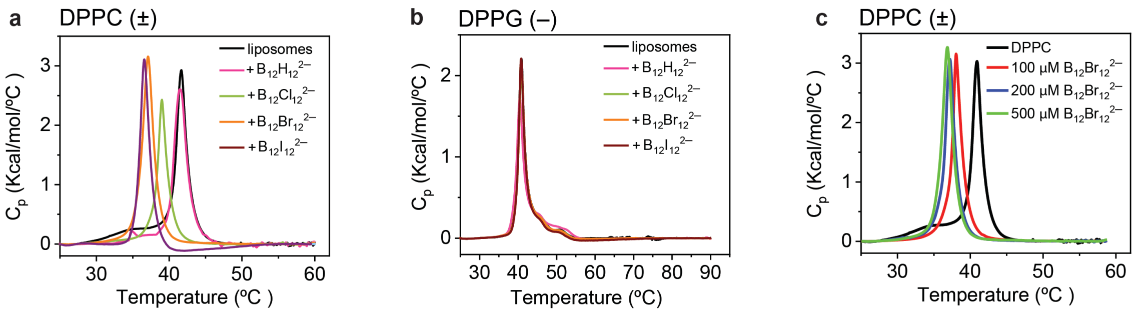

Figure S15: DSC heating thermograms of **a** DPPC and **b** DPPG liposomes (500 µM phospholipid, black line) in the absence and presence of different cluster anions (500 µM); note that POPC was not studied, because it does not show a phase transition in this temperature range, but only at –2°C. **c** DSC heating thermograms of DPPC liposomes (500 µM) alone and with increasing concentrations of $B_{12}Br_{12}^{2-}$.

Table S3: Transition temperatures (T_m), calorimetric (ΔH_t) and van't Hoff (ΔH_{vH}) enthalpies,[a] cooperativity parameter (σ) and the size of cooperative unit (C.U.) of phase transition of DPPC liposomes in the presence of different concentrations of $B_{12}Br_{12}^{2-}$.

	T_m (ºC)	ΔH_t (kcal/mol)[b]	ΔH_{vH} (kcal/mol)[b]	σ (10^{-4})[c]	C.U.[c]
500 µM DPPC	41.7	6.21	331	3.52	53.3
100 µM $B_{12}Br_{12}^{2-}$	38.5	6.16	315	3.82	51.1
200 µM $B_{12}Br_{12}^{2-}$	37.5	6.38	337	3.58	52.8
500 µM $B_{12}Br_{12}^{2-}$	37.1	6.95	394	3.11	56.7

[a] Data fitted with the Levenberg-Marquardt non-linear least-square method, model 2 (non-two-state, see Methods). [b] 5% error (SD, duplicates). [c] 5% error (SD, calculated by considering error propagation with respect both, ΔH_{vH} and ΔH_t).

10. Molecular Dynamics Simulations

Table S4: Force-field parameters[a] for the dodecaborate anion clusters ($B_{12}X_{12}^{2-}$) obtained by using the Seminario method in the VFFDT software.[8]

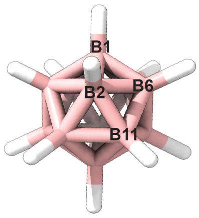

Bond	Parameter	X = H	X = Cl	X = Br	X = I
B–B	r_{eq}[b] (Å)	1.784	1.789	1.790	1.793
	K_r[c] (kcal/mol·Å^2)	78.297	74.961	75.117	71.065
B–X	r_{eq} (Å)	1.202	1.813	1.980	2.214
	K_r (kcal/mol·Å^2)	238.165	153.449	110.850	87.819
**Angle**	**Parameter**	**X = H**	**X = Cl**	**X = Br**	**X = I**
B–B–B[d]	θ_{eq}[e] (°)	60.00	60.00	60.00	60.00
	K_θ[f] (kcal/mol·radian^2)	31.51	29.20	25.54	23.51
B–B–B[g]	θ_{eq} (°)	108.00	108.00	107.99	108.00
	K_θ (kcal/mol·radian^2)	55.90	51.65	44.11	37.64
B–B–X	θ_{eq} (°)	121.71	121.71	121.71	121.71
	K_θ (kcal/mol·radian^2)	36.54	51.00	41.38	38.25
**Nonbonding interaction**	**Parameter**	**X = H**	**X = Cl**	**X = Br**	**X = I**
B	z[h](e)	0.0313	–0.0282	–0.2147	–0.1574
	σ[i] (Å)	4.083	4.083	4.083	4.083
	ε[j] (kcal/mol)	0.180	0.180	0.180	0.180
X	$z(e)$	–0.1979	–0.1383	0.0481	–0.0092
	σ(Å)	1.473	1.945	2.027	2.155
	ε (kcal/mol)	0.016	0.263	0.393	0.495

[a] Dihedral parameters for all clusters were: multiplicity = 1; force constant = 0.0; phase = 0.0; V_{term} = 1. [b] r_{eq}: bond equilibrium distance. [c] K_r: bond force constant. [d] Acute angle between boron atoms e.g. B1-B2-B6 in the structure above table. [e] θ_{eq}: angle. [f] K_θ: angle force constant. [g] Obtuse angle between boron atoms e.g. B1-B2-B11 in the structure above table. [h] Atomic partial charges, calculated at HF/6-31G(d) level of theory[13]. [i] ε: Depth of Lennard-Jones potential. [j] σ: van der Waals radius.

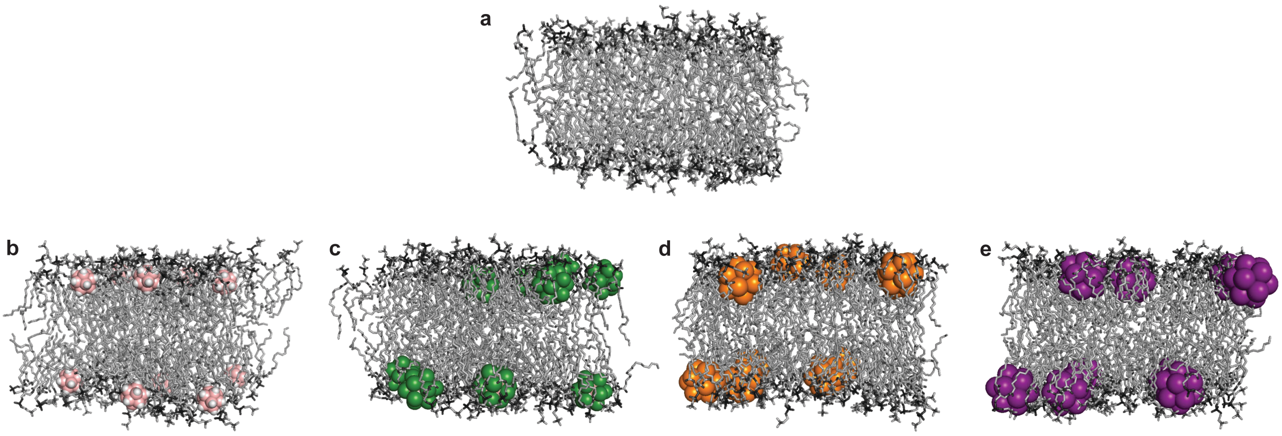

Figure S16: MD simulation snapshots (at 500 ns, 30ºC) of **a** POPC lipid bilayers alone and in the presence of **b** $B_{12}H_{12}^{2-}$, **c** $B_{12}Cl_{12}^{2-}$, **d** $B_{12}Br_{12}^{2-}$, and **e** $B_{12}I_{12}^{2-}$, polar heads; dark grey; hydrophobic tails, light grey; $B_{12}H_{12}^{2-}$; pink; $B_{12}Cl_{12}^{2-}$; green, $B_{12}Br_{12}^{2-}$; orange; $B_{12}I_{12}^{2-}$; purple. Water and sodium counter cations are not shown for clarity.

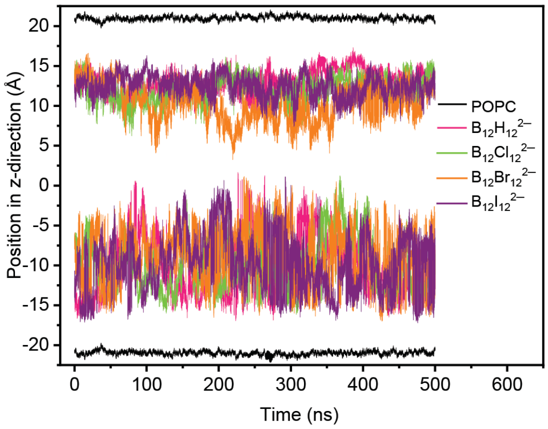

Figure S17: MD-simulated fluctuation profiles through a POPC membrane in the absence (black profile) and presence (colored profiles) of boron clusters (500 ns, 30ºC). Distances were calculated as average values of the center of mass of the boron clusters and of the nitrogen atoms in the lipid headgroup.

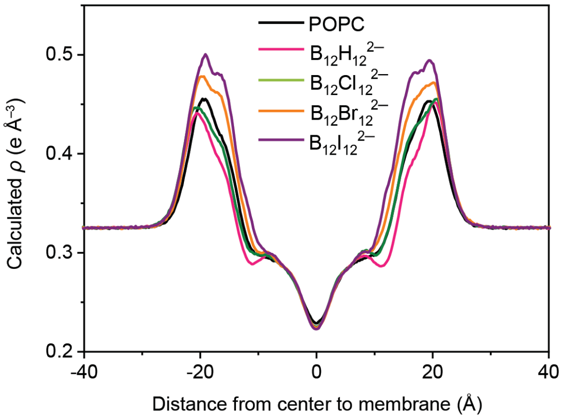

Figure S18: Calculated electron density profiles from MD simulations for a POPC bilayer in the absence (black profile) and presence of boron cluster anions (colored profiles). Note the significant lipid bilayer thinning from 39 Å for neat POPC to 36 Å in the presence of $B_{12}Br_{12}^{2-}$ and 33 Å in the presence of $B_{12}I_{12}^{2-}$, in line with the SAXS measurements (see next section).

11. Small-angle X-ray Scattering

SAXS spectra for samples containing 20 mM POPC (prepared by simple mixing) with increasing $B_{12}I_{12}^{2-}$ concentration are shown in Fig. S19b. Two Bragg peaks arising from the smectic ordering of the multilamellar structure (liposome) are clearly observed at around 0.1 and 0.2 nm^{-1}, corresponding to the 001 and 002 (hkl) reflections and to an interlamellar distance of around 63 Å, in agreement with literature.[23] Upon increasing the $B_{12}I_{12}^{2-}$ concentration, the Bragg peaks vanish, indicating a loss of the smectic ordering, presumably as a result of a “peeling” of the liposome structure, while the interlamellar distance is not affected. The form factor of the bilayers (F_{obs}), observed as a broad oscillation, was fitted with a multi-step function (or box model) of the electron density profile across the bilayer, see Fig. 5 in main text. It is described in Fourier space as a sum of sinusoidal terms as:

$$F_{obs} = \sum_0^n \Delta\rho_n \frac{\sin(qd_n)}{q} \qquad (1)$$

and the scattered intensity is expressed as:

$$I_{box\ model} = a\left(\frac{F_{obs}}{q}\right)^2 \qquad (2)$$

with *a* being a scaling factor.

The fitting parameters for the pure POPC system were initialized with reasonable data ($\Delta\rho_n$ and d_n values, as defined in Fig. 19a) taken from the electron density profile well established in the literature,[24] before the SAXS spectrum was fitted. A good agreement was found between model and experiment (spectrum 20 mM POPC in Fig.

S19b, with a half bilayer thickness d_1 = 22.5 Å, compared to 21.1Å).[25] In the presence of $B_{12}I_{12}^{2-}$, the best fits were obtained by using d_1 and $\Delta\rho_1$ as fitting parameters, resulting in the electron density profiles shown in Fig. 5 in the manuscript. The increase in electron density in the polar head group (integrals) in this figure allowed an estimate of the $B_{12}I_{12}^{2-}$/lipid molar ratios, affording an approximately linear concentration dependence, see Fig. S20.

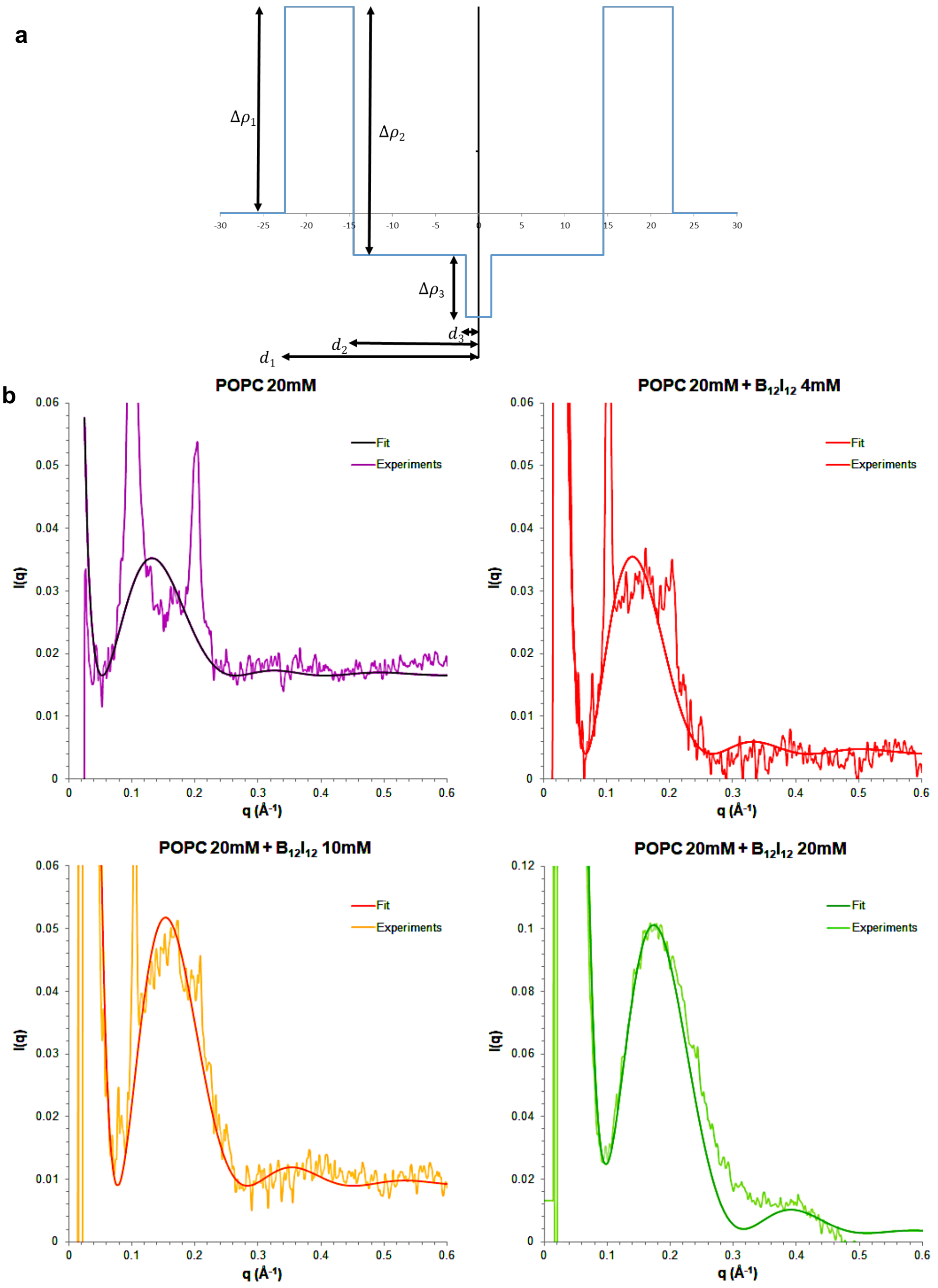

Figure S19: a Schematic electron density profile across the bilayer used for the calculation of the bilayer form factor (F_{obs}), with $\Delta\rho_n$ being the electron density steps (contrast) between each successive step *n,* and d_n being the cumulative distances of the different steps. **b** Experimental and fitted electron density profiles of POPC multilamellar vesicles (20 mM) alone and in the presence of different concentrations of $B_{12}I_{12}^{2-}$.

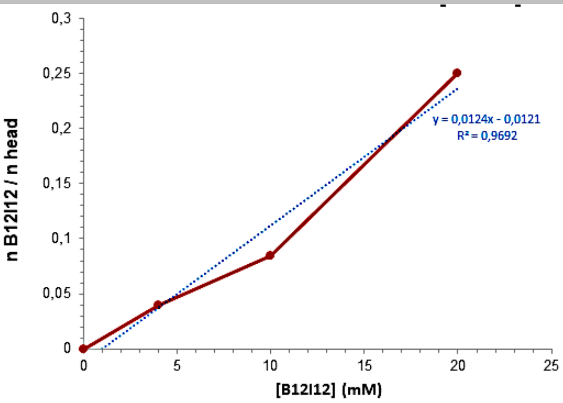

Figure S20: $B_{12}I_{12}^{2-}$/lipid (or lipid polar head) molar ratios in the bilayer calculated from the increase in the electron density in the polar head (see Fig. 5 in main text).

12. References

[1] J. C. M. Stewart, *Anal. Biochem.* **1980**, *104*, 10-14.
[2] A. Blume, *Thermochim. Acta* **1991**, *193*, 299-347.
[3] D. Marsh, *Chem. Phys. Lipids* **1991**, *57*, 109-120.
[4] D. Marsh, A. Watts, P. F. Knowles, *Biochim. Biophys. Acta Biomembr.* **1977**, *465*, 500-514.
[5] S. Jo, T. Kim, V. G. Iyer, W. Im, *J. Comput. Chem.* **2008**, *29*, 1859-1865.
[6] P. Mark, L. Nilsson, *J. Phys. Chem. A* **2001**, *105*, 9954-9960.
[7] C. J. Dickson, R. C. Walker, I. R. Gould, *J. Chem. Theory Comput.* **2022**, *18*, 1726-1736.
[8] S. Zheng, Q. Tang, J. He, S. Du, S. Xu, C. Wang, Y. Xu, F. Lin, *J. Chem. Inf. Model.* **2016**, *56*, 811-818.
[9] B. Kurt, H. Temel, *Chem. Phys. Lett.* **2021**, *775*, 138656.
[10] B. Kurt, H. Temel, *Molecules* **2020**, *25*, 2196.
[11] K. Raghavachari, *Theor. Chem. Acc.* **2000**, *103*, 361-363.
[12] R. Krishnan, J. S. Binkley, R. Seeger, J. A. Pople, *J. Chem. Phys* **1980**, *72*, 650-654.
[13] C. I. Bayly, P. Cieplak, W. Cornell, P. A. Kollman, *J. Phys. Chem.* **1993**, *97*, 10269-10280.
[14] M. e. Frisch, G. Trucks, H. Schlegel, G. Scuseria, M. Robb, J. Cheeseman, G. Scalmani, V. Barone, G. Petersson, H. Nakatsuji, Gaussian, Inc., Wallingford CT, **2016**.
[15] T. Darden, D. York, L. Pedersen, *J. Chem. Phys* **1993**, *98*, 10089-10092.
[16] V. Kräutler, W. F. van Gunsteren, P. H. Hünenberger, *J. Comput. Chem.* **2001**, *22*, 501-508.
[17] D. A. Case, H. M. Aktulga, K. Belfon, D. S. Cerutti, G. A. Cisneros, V. W. D. Cruzeiro, N. Forouzesh, T. J. Giese, A. W. Götz, H. Gohlke, S. Izadi, K. Kasavajhala, M. C. Kaymak, E. King, T. Kurtzman, T.-S. Lee, P. Li, J. Liu, T. Luchko, R. Luo, M. Manathunga, M. R. Machado, H. M. Nguyen, K. A. O'Hearn, A. V. Onufriev, F. Pan, S. Pantano, R. Qi, A. Rahnamoun, A. Risheh, S. Schott-Verdugo, A. Shajan, J. Swails, J. Wang, H. Wei, X. Wu, Y. Wu, S. Zhang, S. Zhao, Q. Zhu, T. E. Cheatham, III, D. R. Roe, A. Roitberg, C. Simmerling, D. M. York, M. C. Nagan, K. M. Merz, Jr., *J. Chem. Inf. Model.* **2023**, *63*, 6183-6191.
[18] D.A. Case, H.M. Aktulga, K. Belfon, I.Y. Ben-Shalom, J.T. Berryman, S.R. Brozell, D.S. Cerutti, I. T.E. Cheatham, G.A. Cisneros, V.W.D. Cruzeiro, T.A. Darden, N. Forouzesh, G. Giambaşu, T. Giese, M.K. Gilson, H. Gohlke, A.W. Goetz, J. Harris, S. Izadi, S.A. Izmailov, K. Kasavajhala, M.C. Kaymak, E. King, A. Kovalenko, T. Kurtzman, T.S. Lee, P. Li, C. Lin, J. Liu, T. Luchko, R. Luo, M. Machado, V. Man, M. Manathunga, K.M. Merz, Y. Miao, O. Mikhailovskii, G. Monard, H. Nguyen, K.A. O'Hearn, A. Onufriev, F. Pan, S. Pantano, R. Qi, A. Rahnamoun, D.R. Roe, A. Roitberg, C. Sagui, S. Schott-Verdugo, A. Shajan, J. Shen, C..L. Simmerling, N.R. Skrynnikov, J. Smith, J. Swails, R.C. Walker, J. Wang, J. Wang, H. Wei, X. Wu, Y. Wu, Y. Xiong, Y. Xue, D.M. York, S. Zhao, Q. Zhu, P. A. Kollman, **2023**.
[19] W. Humphrey, A. Dalke, K. Schulten, *J. Mol. Graph.* **1996**, *14*, 33-38.
[20] L. Schrodinger, *Version* **2015**, *1*, 8.
[21] T. J. Piggot, J. R. Allison, R. B. Sessions, J. W. Essex, *J. Chem. Theory Comput.* **2017**, *13*, 5683-5696.
[22] N. R. Faye, F. Moroté, C. Grauby–Heywang, T. Cohen–Bouhacina, *Int. J. Nanotechnology* **2013**, *10*, 390-403.
[23] H. L. Scott, A. Skinkle, E. G. Kelley, M. N. Waxham, I. Levental, F. A. Heberle, *Biophys. J.* **2019**, *117*, 1381-1386.
[24] S. Tristram-Nagle, H. I. Petrache, J. F. Nagle, *Biophys. J.* **1998**, *75*, 917-925.
[25] D. Pinisetty, D. Moldovan, R. Devireddy, *Ann. Biomed. Eng.* **2006**, *34*, 1442-1451.
